# A Systematic Review of Contrastive Learning in Medical AI: Foundations, Biomedical Modalities, and Future Directions

**DOI:** 10.3390/bioengineering13020176

**Published:** 2026-02-02

**Authors:** George Obaido, Ibomoiye Domor Mienye, Kehinde Aruleba, Chidozie Williams Chukwu, Ebenezer Esenogho, Cameron Modisane

**Affiliations:** 1 Center for Artificial Intelligence and Multidisciplinary Innovations, Department of Auditing, College of Accounting Sciences, University of South Africa, Pretoria 0002, South Africa; emienyid@unisa.ac.za (I.D.M.); arulekd@unisa.ac.za (K.A.); modistc@unisa.ac.za (C.M.); 2 Department of Mathematical Sciences, Georgia Southern University, Statesboro, GA 30460, USA; cchukwu@georgiasouthern.edu

**Keywords:** contrastive learning, self-supervised learning, medical AI, artificial intelligence, representation learning

## Abstract

Medical artificial intelligence (AI) systems depend heavily on high-quality data representations to support accurate prediction, diagnosis, and clinical decision-making. However, the availability of large, well-annotated medical datasets is often constrained by cost, privacy concerns, and the need for expert labeling, motivating growing interest in self-supervised representation learning. Among these approaches, contrastive learning has emerged as one of the most influential paradigms, driving major advances in representation learning across computer vision and natural language processing. This paper presents a comprehensive review of contrastive learning in medical AI, highlighting its theoretical foundations, methodological developments, and practical applications in medical imaging, electronic health records, physiological signal analysis, and genomics. Furthermore, we identify recurring challenges, including pair construction, sensitivity to data augmentations, and inconsistencies in evaluation protocols, while discussing emerging trends such as multimodal alignment, federated learning, and privacy-preserving frameworks. Through a synthesis of current developments and open research directions, this review provides insights to advance data-efficient, reliable, and generalizable medical AI systems.

## 1. Introduction

The increasing digitalization of healthcare has led to an unprecedented accumulation of multimodal medical data, including imaging, clinical records, physiological signals, and genomics [1,2,3,4]. These data provide vast opportunities for artificial intelligence (AI) to improve diagnosis, prognosis, and treatment planning. However, the effectiveness of AI systems in healthcare remains constrained by the scarcity of annotated datasets and the high cost of expert labeling. Medical annotation often requires domain specialists and is subject to inter-observer variability, making the implementation of large-scale supervised learning difficult [5,6,7]. This has stimulated the growing adoption of self-supervised learning (SSL) approaches, which exploit large volumes of unlabeled data to learn meaningful representations that can generalize across downstream tasks with minimal supervision.

Contrastive learning (CL) has become one of the most prominent paradigms within SSL. It operates by comparing data pairs to bring similar instances closer in representation space while pushing dissimilar ones apart [8,9,10,11,12]. Unlike traditional supervised methods, CL relies on instance discrimination and data augmentations to build robust representations without requiring human annotations. Recent studies have demonstrated the effectiveness of CL for medical representation learning by adapting foundational CL frameworks, such as SimCLR, MoCo, BYOL, and SwAV, to domain-specific data characteristics [13,14,15]. For example, Azizi et al. [16] introduced Multi-Instance Contrastive Learning (MICLe) to exploit multiple images per patient case during self-supervised pretraining, improving label efficiency and downstream performance in medical image classification. Sowrirajan et al. [17] proposed MoCo-CXR, a Momentum Contrast adaptation for chest X-ray interpretation, showing improved representation quality and transferability, particularly in low-label regimes and external datasets. Beyond image-only CL, Zhang et al. [18] presented ConVIRT, which aligns medical images with paired radiology reports via a bidirectional contrastive objective, yielding substantial gains in data-efficient learning and retrieval-based evaluation. In physiological signal modeling, Diamant et al. [19] developed Patient Contrastive Learning of Representations (PCLR) for ECGs, using patient identity over time to define positives and demonstrating improved clinical prediction performance across multiple downstream tasks. Consequently, these methods have been extended to medical domains, where data scarcity and heterogeneity remain pressing challenges. The ability of CL to learn invariant and transferable features makes it particularly suitable for applications in medical imaging, electronic health records (EHRs), and multi-omics analysis.

Several recent reviews have examined self-supervised and CL, although their coverage of medical applications is often limited or modality-specific. Jaiswal et al. [20] provided an early survey outlining theoretical principles and algorithmic variants, but did not address domain-specific adaptations for medical data. Gui et al. [21] broadened the scope to include generative and clustering-based self-supervised methods, yet discussion of healthcare contexts remained minimal. Hu et al. [22] presented a comprehensive and systematic survey of CL, summarizing core principles and a universal CL framework, and synthesizing advances across key components such as augmentations, sampling strategies, architectures, and loss functions. Liu [23] reviewed CL for visual representation learning, highlighting key components, limitations, and practical strategies for improving CL pipelines in computer vision. More medically focused reviews include studies, such as Shurrab et al. [24] and several newer works that explicitly target clinical settings. Wang et al. [25] reviewed predictive and contrastive self-supervised learning for medical images, with emphasis on how natural image SSL methods are adapted for medical data. Huang et al. [26] systematically reviewed self-supervised learning for medical image classification across studies published between 2012 and 2022. VanBerlo et al. [27] surveyed evidence on the impact of self-supervised pretraining across imaging modalities, including X-ray, CT, MRI, and ultrasound, with attention to comparisons against supervised baselines and transfer learning protocols. Table 1 summarizes representative review papers on CL, highlighting their scope and coverage of medical applications.

In contrast to prior reviews that primarily emphasize general CL foundations or focus narrowly on medical imaging, this review provides a cross-modality synthesis spanning medical imaging, EHRs, physiological signals, genomics and proteomics, and multimodal vision language systems. We further emphasize clinically grounded design choices for medical CL, including pairing strategies, augmentation validity, evaluation regimes, and reporting requirements for reproducibility and external validation.

While informative, these studies share common limitations. They often restrict attention to a single modality, typically medical imaging, provide limited cross-modality synthesis, and lack an operational taxonomy that clearly links CL design choices to clinical data characteristics. In addition, evaluation practices are frequently underanalyzed, with insufficient emphasis on external validation, robustness, reproducibility, and cross-domain generalization. To address these gaps, this paper provides a comprehensive review of CL in medical AI, covering its theoretical foundations, methodological advances, and practical applications across diverse biomedical data modalities. The main contributions of this study are as follows:

- We propose an operational, domain-aware taxonomy of medical CL methods grounded in core design components, including loss functions, positive and negative pairing strategies, augmentation policies, and evaluation regimes.
- We synthesize CL applications across key medical modalities, including medical imaging, electronic health records, physiological time series, genomics and proteomics, and multimodal vision language systems, highlighting modality-specific challenges and transferable design patterns.
- We critically examine methodological limitations in the literature, with emphasis on evaluation heterogeneity, reproducibility constraints, data access limitations, and the need for standardized benchmarking and external validation.
- We provide practical guidance and prioritized future directions for clinically trustworthy CL, focusing on robustness to distribution shift, interpretability, fairness, privacy-preserving training, and deployment considerations.

The remainder of the paper is organized as follows. Section 2 presents the methodology of this review. Section 3 describes the foundations of CL. Section 4 reviews applications across medical imaging, electronic health records, genomics and proteomics, multimodal learning, and physiological signal analysis. Section 5 discusses key challenges and limitations. Section 6 outlines future research directions, and Section 7 concludes the paper.

## 2. Methodology

We conducted a systematic review to identify and synthesize CL methods applied to medical and biomedical data. The review question was “How are CL objectives designed and evaluated across medical modalities, and what methodological practices enable robust, reproducible, and clinically meaningful performance?”

### 2.1. Databases and Search Strategy

We searched PubMed, IEEE Xplore, ACM Digital Library, Web of Science, Scopus, and arXiv for studies published between January 2019 and October 2025. The time window captures the maturation of modern CL frameworks and their adoption in medical AI. Searches were executed on 31 October 2025.

Search queries combined contrastive/self-supervised learning terms with medical domain keywords. Representative query patterns included:

- (“contrastive learning” OR “self-supervised”) AND (medical OR healthcare OR clinical)
- (“contrastive learning” OR SimCLR OR MoCo OR BYOL) AND (radiology OR “chest x-ray” OR MRI OR CT)
- (“contrastive learning” OR “self-supervised”) AND (“electronic health record” OR EHR OR “clinical notes”)
- (“contrastive learning” OR “representation learning”) AND (ECG OR EEG OR “physiological signals”)
- (“contrastive learning” OR “self-supervised”) AND (genomics OR proteomics OR “single-cell”)

Search strings were adapted to database-specific syntax and indexing conventions. Backward and forward citation chasing was performed for key studies to identify additional relevant articles.

### 2.2. Eligibility Criteria

We included:

- Peer-reviewed journal articles and full conference papers; influential preprints were included selectively when they introduced widely adopted methods, benchmarks, or were heavily cited in subsequent peer-reviewed work.
- Studies that explicitly employed CL or closely related objectives (e.g., InfoNCE, supervised contrastive loss, MoCo/SimCLR/BYOL-style frameworks, CLIP-style alignment).
- Studies using medical or biomedical data (medical imaging, EHRs, physiological time series, genomics/proteomics, pathology, or multimodal combinations).

We excluded:

- Non-medical studies without biomedical datasets or clinically motivated tasks.
- Abstract-only records, posters lacking sufficient methodological detail, editorials, commentaries, and theses.
- Non-validated technical reports and preprints without experimental results, or preprints superseded by peer-reviewed versions.

When multiple papers described incremental versions of the same method, we prioritized the most comprehensive peer-reviewed version and retained earlier versions for historical context when needed.

### 2.3. Study Selection

**Figure 1:**
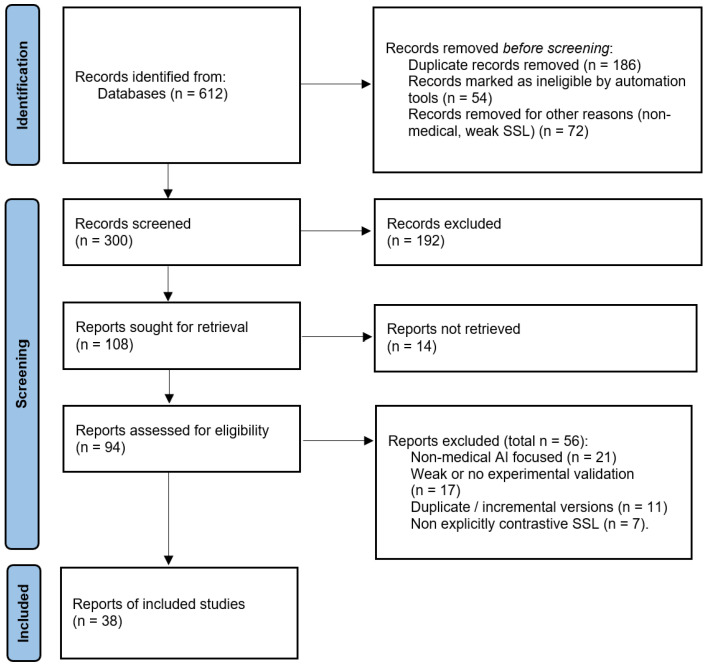
PRISMA diagram of the literature selection process.

Records were deduplicated, then screened by title/abstract, followed by full-text assessment using the eligibility criteria. Screening was performed independently by G.O. and I.D.M. Disagreements were resolved by consensus, and unresolved conflicts were adjudicated by K.A., E.E., C.M., and C.W.C. Reasons for full-text exclusion were recorded (e.g., non-medical domain, insufficient methodological detail, no contrastive objective, no empirical evaluation). Of 612 identified records, 300 were screened after duplicate and automated exclusions. Following a full-text assessment of 94 reports, 38 studies were included. Figure 1 summarizes the selection process.

### 2.4. Data Extraction

We extracted study characteristics using a standardized template and cross-checked entries for consistency. For each study, we recorded the following:

- Modality and dataset(s);
- Contrastive formulation (loss, supervision regime, pairing strategy, negative sampling);
- Encoder architecture and pretraining scale;
- Downstream task(s), evaluation regime (linear probe, fine-tuning, few-shot/zero-shot), and metric(s);
- Reported limitations, external validation, and reproducibility artifacts (e.g., code availability).

### 2.5. Taxonomy of Medical Contrastive Learning

To consolidate the heterogeneous methodological landscape of medical CL, we introduce a taxonomy that groups approaches according to a small set of design dimensions that materially affect (i) the clinical invariances encoded in learned representations, (ii) the source and strength of supervision used during pretraining, and (iii) the evidentiary standard used to claim downstream benefit. The goal of this taxonomy is twofold. First, it provides a consistent analytic framework for comparing studies across modalities and clinical endpoints. Second, it serves as a reporting scaffold by making explicit the minimum set of methodological decisions that must be specified to support reproducibility and meaningful clinical interpretation.

Table 2 summarizes the taxonomy dimensions applied throughout this manuscript. The loss family or objective defines the learning signal and distinguishes objectives that rely on explicit negatives, such as InfoNCE or NT-Xent, incorporate labels during pretraining through supervised CL, adopt negative-free teacher–student distillation, or implement clustering-based consistency. The pairing strategy specifies what constitutes a positive relation in clinical data, including augmentations of the same instance, same-class positives, temporally adjacent windows, and patient-level correspondences across time. This choice is particularly consequential in medical datasets because cohort structure, repeated measures, and phenotype similarity can lead to false negatives and shortcut learning unless pairing rules are explicitly controlled. Augmentation and view design operationalize invariances and should be clinically plausible, since modality-inappropriate transformations may suppress pathology in imaging, distort physiological rhythms in biosignals, or alter semantic content in clinical text.

The label regime captures the extent of supervision used during representation learning and distinguishes unsupervised settings from weakly supervised, semi-supervised, and fully supervised pretraining. This distinction is essential for fair comparison because label access during pretraining changes both the information content of the learning signal and the interpretation of label-efficiency gains. The evaluation protocol defines how representation quality is assessed, including the downstream training regime, linear probing versus fine-tuning, evaluation under label scarcity through label-fraction sweeps, and testing under clinically relevant distribution shifts such as temporal, site, scanner, or device, and demographic shift. Calibration and uncertainty reporting are also important when risk prediction is a primary endpoint. Finally, studies are grouped by task family, including classification, segmentation, retrieval, and prognosis, since both contrastive design choices and evaluation expectations are strongly task-dependent and should be compared under aligned clinical objectives.

### 2.6. Quality and Reporting Appraisal

Because medical CL studies vary widely in design and reporting, we used a lightweight methodological checklist focused on reproducibility and clinical validity (Table A1; Appendix B). The checklist was used to characterize the evidence base and identify common gaps; it was not used to exclude studies.

### 2.7. Protocol Registration

No protocol was preregistered. This review was conducted to map and synthesize rapidly evolving methodological literature. To support transparency and reproducibility, we provide database-specific search strategies (Appendix A) and an explicit appraisal checklist (Table A1; Appendix B).

## 3. Overview of Contrastive Learning

**Figure 2:**
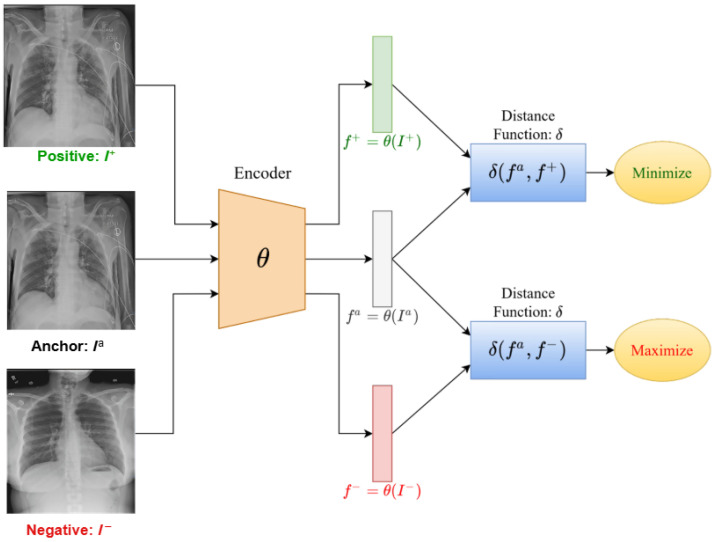
Steps in the CL process, adapted from [39].

CL is a self-supervised learning technique designed to learn effective representations from unlabeled data by distinguishing between positive and negative pairs [11,22,28,29,30]. Unlike traditional supervised learning, which heavily depends on labeled data to guide the learning process, CL utilizes the intrinsic structure of the data to derive semantically meaningful representations [20,21,31,32]. This makes it especially effective in situations where labeled data is limited or difficult to obtain. The concept of CL originated from the broader field of self-supervised learning, a paradigm that has seen increasing interest due to its ability to leverage vast amounts of unlabeled data. The early developments of CL date back to the 1990s with the introduction of metric learning and Siamese networks, which were designed to learn similarity metrics between pairs of data points [33]. These early forms laid the groundwork for the evolution of CL into a robust tool for modern machine learning applications. Figure 2 illustrates the general workflow of CL, in which an encoder parameterized by θ maps an anchor, a positive, and a negative example into a shared embedding space. The goal is to maximize similarity between the anchor and positive pair while minimizing similarity to the negative.

Originally introduced for verification tasks using Siamese networks, contrastive objectives gained traction through contrastive predictive coding (CPC) and frameworks, such as SimCLR and MoCo, which demonstrated that high-quality representations could be learned from unlabeled data at scale [20,34,35,36]. These advances catalyzed rapid adoption across fields including medical imaging, text analysis, and multimodal learning [37,38].

Furthermore, the theoretical foundations of CL are rooted in the idea of representation learning through similarity and dissimilarity. The main goal is to learn a function $f_{\theta }$ that maps input data points to a representation space where semantically similar inputs are closer together, while semantically dissimilar inputs are further apart [21]. This is operationalized through a contrastive loss function, commonly referred to as Noise Contrastive Estimation (InfoNCE) loss. The InfoNCE loss is defined as follows:

(1) $$L=-\log\frac{\exp(\mathrm{sim}\left(z_{i},z_{j}\right)/\tau )}{\sum_{k=1}^{K}\exp\left(\mathrm{sim}\left(z_{i},z_{k}\right)/\tau \right)},$$

where $z_{i}$ and $z_{j}$ are the embeddings of the anchor and the positive sample, $\mathrm{sim}(z_{i},z_{j})$ denotes the similarity between two embeddings, typically calculated using cosine similarity: $\mathrm{sim}\left(z_{i},z_{j}\right)=\frac{z_{i}\cdot z_{j}}{∥z_{i}∥∥z_{j}∥}$, τ is a temperature parameter that regulates the sharpness of the similarity distribution, and the denominator sums over all *K* samples in the dataset, encompassing both positive and negative pairs.

This loss function seeks to maximize the similarity between the embeddings of positive pairs while minimizing the similarity between the anchor and negative samples [40,41]. The efficacy of CL is determined by several key factors. The selection of positive and negative pairs is critical, as it directly influences the quality of the learned representations. For instance, utilizing multiple views or augmentations of the same data point as positive pairs can help the model learn invariances to specific transformations [42]. Additionally, the use of larger batch sizes or memory banks can provide the model with a richer set of negative samples, enhancing the learning process. The choice of data augmentation strategies is also vital, as it determines the types of invariances the model will learn.

### 3.1. Variants and Extensions of Contrastive Learning

To address practical limitations such as negative sampling bias, augmentation sensitivity, modality heterogeneity, and temporal dependence, CL has evolved into several complementary variants. In medical AI, these variants differ primarily in whether they rely on explicit negatives, how positives are constructed (instance-level vs patient-level), and whether the objective aligns representations within a single modality or across modalities.

#### 3.1.1. InfoNCE-Based Contrastive Learning (SimCLR, MoCo, SupCon)

The most widely used family of methods is based on the InfoNCE objective, which maximizes similarity between positive pairs while contrasting them against a set of negatives. SimCLR forms positive pairs using two augmented views of the same sample and relies on large batch sizes to provide many in-batch negatives [43]. Its loss can be written as follows:

(2) $$L_{\mathrm{SimCLR}}=\sum_{i\in I}-\log\frac{\exp(\mathrm{sim}\left(z_{i},z_{j}\right)/\tau )}{\sum_{k=1}^{2N}{⊮}_{\left[k\neq i\right]}\exp\left(\mathrm{sim}\left(z_{i},z_{k}\right)/\tau \right)}$$

SimCLR relies on carefully designed stochastic augmentations to define positive pairs, meaning that the choice of augmentation policy strongly determines what invariances are learned [44,45,46]. In medical imaging, this is particularly important because aggressive transformations, such as heavy cropping, blurring, or color jitter, can remove or distort subtle pathology signals and may unintentionally encourage shortcut learning. Because SimCLR treats all other samples in the minibatch as negatives, it benefits substantially from large batch sizes, which increase the number and diversity of in-batch negatives and improve representation quality. However, this assumption can be problematic in clinical datasets where semantically similar cases, such as patients sharing the same diagnosis or repeated examinations from related cohorts, may appear in the same batch, creating false negatives that reduce downstream performance and calibration.

MoCo improves scalability by maintaining a queue (memory bank) of negative keys and using a momentum encoder to stabilize feature representations across iterations [47,48,49]. The MoCo loss function is defined similarly to the InfoNCE loss but incorporates a momentum encoder:

(3) $$L_{\mathrm{MoCo}}=-\log\frac{\exp(\mathrm{sim}\left(q,k^{+}\right)/\tau )}{\sum_{i=0}^{K}\exp\left(\mathrm{sim}\left(q,k_{i}\right)/\tau \right)}$$

where *q* is the query embedding from the current batch, $k^{+}$ is the key embedding of the positive sample, $k_{i}$ are the embeddings of negative samples stored in the memory bank, and τ is the temperature parameter [47,50,51]. Through maintaining a queue of negative samples and using a slowly updated encoder, MoCo effectively improves the model’s ability to learn discriminative features.

Supervised Contrastive Learning (SupCon) extends the same principle to labeled settings by treating all samples from the same class as positives [29]. This formulation is relevant to medical AI for fine-tuning and hybrid training regimes where limited labels are available:

(4) $$L_{\sup}=\sum_{i\in I}\frac{-1}{\left|P(i)\right|}\sum_{p\in P(i)}\log\frac{\exp(\mathrm{sim}\left(z_{i},z_{p}\right)/\tau )}{\sum_{a\in A(i)}\exp\left(\mathrm{sim}\left(z_{i},z_{a}\right)/\tau \right)}.$$

Although effective, InfoNCE-based methods can suffer from false negatives and batch-size dependence, which are amplified in medical datasets where patients may share similar phenotypes or repeated examinations.

#### 3.1.2. Negative-Free Self-Distillation (BYOL, DINO, SimSiam)

A second major family of approaches removes explicit negative samples and instead relies on self-distillation or cross-view prediction between augmented views. This design is particularly appealing for medical AI, where (i) batch sizes are often constrained by high-resolution imaging or long physiological sequences, (ii) datasets are highly imbalanced, and (iii) many samples can be semantically similar due to shared diagnoses, repeated examinations, or cohort effects. In such settings, InfoNCE-style negative sampling can introduce harmful false negatives, weakening representation quality and downstream calibration.

Bootstrap Your Own Latent (BYOL) learns representations using an online network that predicts the embedding produced by a slowly evolving target network, with the target parameters updated via exponential moving average [52,53,54]. By eliminating dependence on large numbers of negatives, BYOL reduces the need for very large batches and can be more stable under clinical class imbalance and limited-label regimes.

DINO (Self-Distillation with No Labels) similarly adopts a teacher–student paradigm, training the student to match the teacher’s output distribution under different augmentations [55,56,57,58]. The objective is typically defined as a cross-entropy loss between teacher and student predictions:

(5) $$L_{\mathrm{DINO}}=-\sum_{x\in X}p_{\mathrm{teacher}}\left(x\right)\log p_{\mathrm{student}}\left(x\right).$$

DINO has shown strong performance in representation learning and is often used in medical imaging pipelines where interpretability-relevant attention maps and robust global features are desired.

SimSiam learns representations by predicting one augmented view from another while employing stop-gradient operations to prevent collapse [59,60,61,62]. In biomedical settings, negative-free objectives are especially useful for learning invariances in modalities such as histopathology, radiology, ECG, and EEG, where clinically meaningful similarity can occur across patients and where treating similar cases as negatives may degrade transfer performance. Overall, negative-free self-distillation provides a practical alternative to InfoNCE-based CL when negative sampling is unreliable or batch scaling is infeasible.

#### 3.1.3. Clustering-Based Self-Supervised Learning (SwAV)

Clustering-based CL replaces explicit pairwise instance discrimination with a clustering objective. SwAV (Swapping Assignments between Views) performs online clustering and encourages consistent cluster assignments across augmentations [63]. This can reduce dependence on large numbers of negatives and may better preserve subtle pathology features by avoiding overly aggressive augmentation policies. Such methods are relevant in medical imaging, where representation stability and texture-level features are critical.

#### 3.1.4. Multimodal Contrastive Alignment (CLIP, ConVIRT, BioViL)

CL has been extended beyond single-modality learning to align heterogeneous biomedical modalities into a shared embedding space. The most influential paradigm is CLIP-style vision–language pretraining, which trains an image encoder and a text encoder jointly using paired image–text data [64]. Given a minibatch of *N* paired samples ${\left\{\left(x_{i},t_{i}\right)\right\}}_{i=1}^{N}$, encoders produce normalized embeddings $z_{i}^{I}$ and $z_{i}^{T}$. Training maximizes similarity for matched pairs and minimizes similarity for mismatched pairs using a bidirectional retrieval objective (image-to-text and text-to-image), typically implemented as symmetric cross-entropy over in-batch negatives:

(6) $$L_{\mathrm{CLIP}}=\frac{1}{2}\left(-\frac{1}{N}\sum_{i=1}^{N}\log\frac{\exp(\langle z_{i}^{I},z_{i}^{T}\rangle /\tau )}{\sum_{j=1}^{N}\exp\left(\langle z_{i}^{I},z_{j}^{T}\rangle /\tau \right)}-\frac{1}{N}\sum_{i=1}^{N}\log\frac{\exp(\langle z_{i}^{T},z_{i}^{I}\rangle /\tau )}{\sum_{j=1}^{N}\exp\left(\langle z_{i}^{T},z_{j}^{I}\rangle /\tau \right)}\right),$$

where τ is a temperature parameter and 〈·,·〉 denotes cosine similarity. This formulation produces aligned multimodal representations that support retrieval and prompt-based zero-shot transfer: downstream classification can be performed by comparing an image embedding with text embeddings of label prompts (e.g., “no pleural effusion” vs “pleural effusion”), enabling label-free inference.

In medical AI, CLIP-style objectives have enabled major advances in radiology and pathology by leveraging radiology reports or biomedical captions as weak supervision. ConVIRT aligned chest X-rays with paired radiology reports via bidirectional CL, improving label efficiency and retrieval-based evaluation [18]. Subsequent work strengthened alignment through finer-grained supervision: GLoRIA introduced global–local alignment between image regions and report phrases to improve grounding and reduce spurious correlations [65]. BioViL further improved semantics by incorporating biomedical language pretraining, yielding stronger transferable representations and improved zero-shot performance [66]. Extensions, such as BioViL-T, incorporate temporal alignment across prior and current studies, improving progression-sensitive recognition [67]. Prompt-based adaptations, such as CXR-CLIP, integrate radiologist-defined class prompts to enhance clinical interpretability and reduce label ambiguity [68]. Zero-shot clinical deployment has also been explored through CLIP-based radiology models such as CheXzero [69].

A central limitation in clinical CLIP-style learning is that reports are noisy supervision: they contain negation (e.g., “no pneumothorax”), uncertainty (e.g., “cannot exclude”), templated phrases, and study-level context that may not map cleanly to image-level findings. Misalignment can create false negatives (a finding present in the image but unmentioned in the text) and shortcut learning driven by site, protocol, or demographics. Consequently, medical variants often incorporate filtering, entity extraction, uncertainty handling, or knowledge-aware objectives. For example, MedCLIP reduces dependency on strictly paired data by decoupling image and text corpora and using knowledge-enhanced supervision, improving robustness under limited or noisy pairings [70]. Beyond radiology, pathology vision–language models such as PLIP and CONCH scale CLIP-style pretraining to histopathology captions, enabling strong zero-shot transfer across datasets and tasks [71,72]. Large biomedical foundation models such as BiomedCLIP and PMC-CLIP further extend multimodal contrastive alignment by leveraging literature-scale figure–caption corpora to support broad biomedical retrieval, transfer, and few-shot adaptation [73,74].

#### 3.1.5. Temporal and Patient-Aware Objectives (CPC, CLOCS/PCLR)

Medical data often exhibit sequential structure and repeated measurements, motivating contrastive objectives that exploit temporal and patient-identity information. Contrastive Predictive Coding (CPC) learns representations by predicting future latent embeddings in a sequence, using a contrastive objective to distinguish true future representations from negatives [75]. CPC is well-suited to physiological signals such as ECG and EEG, where long-range temporal dependence is clinically meaningful.

Patient-aware extensions define positives using identity or longitudinal structure. For example, Patient Contrastive Learning of Representations (PCLR) treats ECG recordings from the same patient as positives and recordings from different patients as negatives, improving generalization across downstream clinical prediction tasks [19]. Similarly, CLOCS introduces spatiotemporal contrastive structure by aligning signals across time and across leads, improving robustness under lead variations and temporal drift [76]. These objectives are particularly relevant in medicine, where patient-level consistency and longitudinal trajectories are central to clinical decision-making.

#### 3.1.6. Optimization Refinements (Hard Negatives, Debiased Objectives)

Medical datasets frequently contain semantically similar cases, repeated examinations, and cohort effects, which makes naive negative sampling prone to false negatives. This issue is especially acute in clinical cohorts where different patients may share the same diagnosis or phenotype, and where repeated studies from the same hospital or scanner can introduce hidden correlations. Hard negative mining and debiased contrastive losses mitigate these issues by reweighting negatives, correcting sampling bias, or explicitly filtering likely false negatives [77,78].

In practice, hard negative mining prioritizes negatives that are close to the anchor in representation space (i.e., most confusing samples), which can sharpen decision boundaries but may also amplify errors if hard negatives are actually clinically similar positives. Debiased objectives address the mismatch between the InfoNCE assumption that all negatives are truly dissimilar and real-world medical data, where batch negatives may contain unobserved positives due to label scarcity, weak supervision, and reporting noise. These refinements improve robustness under clinical heterogeneity and reduce representation shortcuts driven by site, protocol, demographic confounders, or disease prevalence patterns.

A common refinement is to apply weights to negatives in the denominator of InfoNCE, emphasizing harder (more similar) negatives:

(7) $$L_{\mathrm{HN}}=-\log\frac{\exp(\mathrm{sim}\left(z,z^{+}\right)/\tau )}{\exp\left(\mathrm{sim}\left(z,z^{+}\right)/\tau \right)+\sum_{k=1}^{K}w_{k}\;\exp\left(\mathrm{sim}\left(z,z_{k}^{-}\right)/\tau \right)}$$

where $w_{k}$ increases with similarity (e.g., $w_{k}\propto \exp\left(\mathrm{sim}\left(z,z_{k}^{-}\right)/\beta \right)$) so that negatives closer to the anchor contribute more strongly. In medical data, this strategy must be used cautiously because clinically similar cases may be incorrectly treated as negatives.

To reduce the impact of false negatives, debiased CL modifies the negative term by accounting for the probability that some negatives are actually positives. Following the debiased contrastive objective [77,79,80], the loss can be written as follows:

(8) $$L_{\mathrm{Debiased}}=-\log\frac{\exp(\mathrm{sim}\left(z,z^{+}\right)/\tau )}{\exp\left(\mathrm{sim}\left(z,z^{+}\right)/\tau \right)+K\cdot {\tilde{p}}_{\mathrm{neg}}}$$

where ${\tilde{p}}_{\mathrm{neg}}$ is a corrected negative expectation term that subtracts the estimated contribution of false negatives. This formulation is particularly relevant in medical AI because minibatches may contain semantically similar patients (shared condition, demographic similarity, repeated study types), even when labels are missing or incomplete [81,82,83].

### 3.2. Datasets and Benchmarks for Medical Contrastive Learning

A key barrier to fair comparison in medical CL is the diversity of datasets, label taxonomies, and evaluation protocols used across modalities. To improve reproducibility and enable more meaningful benchmarking, we summarize widely used public datasets and community benchmarks for contrastive and self-supervised representation learning in medical AI, spanning imaging, EHRs, physiological signals, and omics. We also highlight common evaluation protocols (linear probing, fine-tuning, few-shot transfer, external validation) and task-appropriate metrics that recur across the literature. Table 3 presents representative public datasets and community benchmarks commonly used in medical CL, grouped by modality, together with typical downstream tasks used for evaluation.

Chest radiography is the most common benchmark for medical CL because it provides large-scale paired image–report corpora and standardized multi-label tasks. Prominent resources include MIMIC-CXR (images with reports) [84] and CheXpert [85], alongside earlier large-scale X-ray datasets such as NIH ChestX-ray14 [86]. Beyond X-rays, neuroimaging benchmarks such as ADNI support Alzheimer’s disease research and multimodal clinical studies [87]. For segmentation and structured imaging tasks, community challenges such as BraTS (brain tumor MRI segmentation) provide standardized datasets, splits, and metrics [88,89]. Dermatology imaging is frequently benchmarked via the ISIC Archive and its associated tasks [90]. In computational pathology, whole-slide image benchmarks and paired resources have grown rapidly, including CAMELYON-style lymph node metastasis benchmarks and large-scale cancer slide repositories derived from TCGA and related infrastructures [91,92].

EHR-based CL often builds patient representations from longitudinal visits and mixed structured/unstructured data. The most widely used public critical-care benchmarks are MIMIC-III [93] and MIMIC-IV [94], with complementary ICU cohorts such as the eICU Collaborative Research Database [95]. These datasets support mortality prediction, length-of-stay prediction, readmission risk, phenotyping, and treatment trajectory modeling, typically under strong temporal and site-specific confounding.

Self-supervised and CL on physiological signals are commonly evaluated on ECG and EEG corpora with large unlabeled volumes and clinically meaningful downstream tasks. PTB-XL is a standard ECG benchmark supporting multi-label ECG classification and transfer learning [96]. EEG benchmarks often use large clinical corpora, such as the TUH EEG dataset for seizure detection and broader EEG event modeling [97].

Omics benchmarks include bulk genomics and transcriptomics resources (e.g., TCGA [98]) and tissue-specific expression atlases (e.g., GTEx [99]). Large biobanks, such as UK Biobank, further enable genotype–phenotype analysis for representation learning and risk modeling [100]. In parallel, single-cell transcriptomics has become a prominent benchmark for CL because of its sparsity and strong batch effects, supported by atlas-scale efforts and curated reference datasets [101].

**Table 3:** Representative public datasets and benchmarks commonly used in medical contrastive learning, grouped by modality.

Modality	Representative Datasets/Benchmarks	Typical Downstream Tasks
Chest imaging	MIMIC-CXR [84]; CheXpert [85]; NIH ChestX-ray14 [86]	Multi-label classification (AUROC); label-scarce transfer; external validation; retrieval and zero-shot classification
General medical imaging	BraTS [88,89]; ISIC Archive [90]; ADNI [87]	Segmentation (Dice/HD95); lesion classification; progression prediction; robustness across scanners and time
Computational pathology	CAMELYON [91,92]; TCGA-derived slide repositories [98]	WSI classification; region retrieval; weakly supervised detection; cross-site generalization
EHR (clinical records)	MIMIC-III [93]; MIMIC-IV [94]; eICU [95]	Mortality and LOS prediction; readmission; phenotyping; temporal outcome modeling; multimodal fusion with notes
Physiological signals	PTB-XL [96]; TUH EEG [97]	Diagnosis classification; seizure detection; patient-level transfer; low-label training
Genomics and transcriptomics	TCGA [98]; GTEx [99]; UK Biobank [100]	Subtype prediction; survival and risk modeling; representation transfer across cohorts; cross-tissue generalization
Single-cell omics	Human Cell Atlas [101]	Cell-type clustering; batch correction; rare cell detection; perturbation response prediction

Across modalities, medical CL is typically evaluated under one or more of the following regimes: (i) linear probing, where the encoder is frozen, and a lightweight classifier is trained to test representation quality under minimal supervision; (ii) full fine-tuning, where the pretrained encoder is adapted end-to-end to measure downstream task performance under realistic clinical training conditions; (iii) few-shot or label-scarce transfer, which evaluates label efficiency by measuring performance as a function of annotated data fraction, such as 1%, 10%, and 100%; and (iv) external validation, where models are evaluated across institutions, scanners, devices, acquisition protocols, or time periods to quantify robustness to domain shift and distribution drift.

Evaluation metrics are task dependent [102,103,104]. For multi-label classification in imaging, particularly chest radiography, performance is commonly reported using the area under the receiver operating characteristic curve (AUROC), and the area under the precision–recall curve (AUPRC), often complemented by macro-F1 or clinically meaningful sensitivity and specificity at fixed thresholds. In segmentation tasks, overlap and boundary-based measures, such as the Dice coefficient and the 95th percentile Hausdorff distance (HD95), are widely used. For prediction tasks on electronic health records and physiological signals, patient-level discrimination metrics such as AUROC and AUPRC are frequently reported, and several studies additionally include calibration metrics such as the Brier score or expected calibration error to assess whether probabilistic outputs are clinically reliable. For vision–language models, benchmarking extends beyond classification to evaluate multimodal alignment and grounding. Common benchmarks include cross-modal retrieval using Recall@K and median rank, phrase grounding scores, and zero-shot classification using prompt embeddings that map clinical labels into the text representation space. Importantly, medical CL studies increasingly distinguish between representation evaluation protocols such as linear probing and deployment-relevant protocols such as full fine-tuning and external validation, since gains under frozen-feature testing do not necessarily translate to robustness, calibration, or clinical reliability in real-world workflows. Table 4 presents common benchmarking protocols and evaluation metrics used to assess medical CL models across modalities, including linear probing, full fine-tuning, few-shot transfer, and external validation under distribution shift.

## 4. Applications of Contrastive Learning in Medical AI

**Figure 3:**
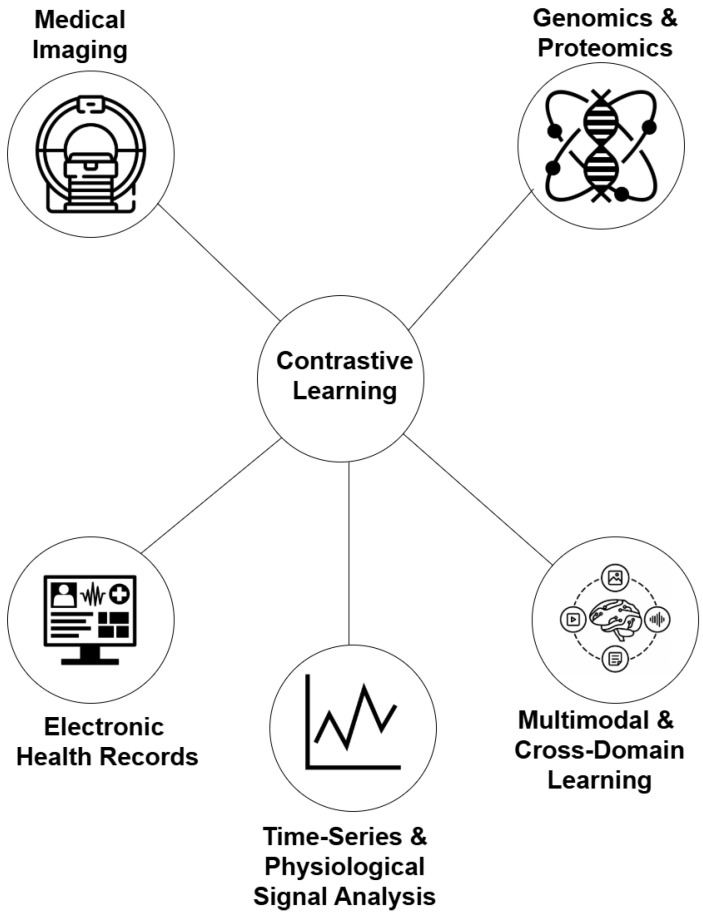
Application Areas of Contrastive Learning in Medical AI.

CL has shown significant promise in the field of medical AI due to its ability to learn effective representations from limited or unlabeled data. This capability is particularly valuable in medical contexts, where labeled data can be scarce or expensive to obtain. Figure 3 presents several applications of CL in healthcare. The following subsections explore the applications of CL across various domains within medical AI. A full summary of the included studies is presented in Appendix C.

### 4.1. Medical Imaging

Medical imaging is one of the most prominent areas where CL has been applied successfully. Clinical imaging pipelines commonly target disease classification, lesion detection, organ or tumor segmentation, retrieval, and anomaly detection. However, robust model development is often limited by the scarcity of high-quality expert annotations, inter-observer variability, and substantial heterogeneity across scanners, acquisition protocols, and institutions. CL addresses these challenges by leveraging large-scale unlabeled imaging repositories to learn transferable representations, improving label efficiency, and supporting more reliable generalization across clinical settings.

A common strategy is to pretrain encoders on unlabeled images using contrastive objectives that enforce invariance across augmentations while preserving clinically meaningful structure. For example, Azizi et al. [16] applied CL to medical imaging tasks such as classification and segmentation by learning representations from unlabeled medical images. They utilized SimCLR to pretrain a model on a large dataset of unlabeled chest X-rays and then fine-tuned the encoder on smaller labeled datasets for downstream tasks. Their results demonstrated consistent performance gains compared to training from scratch, highlighting the value of contrastive pretraining in low-label medical imaging scenarios.

Beyond fully self-supervised pretraining, CL has also been adopted for semi-supervised imaging pipelines. Chaitanya et al. [105] developed a CL approach for semi-supervised learning in medical imaging, with the objective of improving robustness under limited labels and anatomical variability. Their method learns consistent representations across augmented views of the same image as positive pairs while contrasting against other images as negatives, leading to improved performance in MRI classification and segmentation tasks.

Histopathology has emerged as another high-impact imaging domain for CL due to the scale and complexity of whole-slide images and the cost of expert annotations. Ciga et al. [106] explored self-supervised CL for histopathological image analysis and showed that the learned representations could distinguish tissue patterns and malignancy-related morphology effectively. Their findings suggest that contrastive objectives can capture subtle texture and micro-structural cues important for cancer detection and grading, even when labeled samples are limited.

CL has also been tailored to dense prediction tasks such as segmentation, where structural consistency and multi-scale context are essential. Guo et al. [107] proposed a CL framework for cardiac MRI segmentation that incorporates a multi-scale contrastive loss to learn representations at different spatial resolutions. This design supports learning both global anatomical structures and local pathological variations. Their results demonstrated improved segmentation accuracy for cardiac structures such as the myocardium and ventricles, illustrating the usefulness of contrastive objectives for robust segmentation in heterogeneous imaging data.

In addition to supervised and semi-supervised pipelines, CL has been applied to unsupervised anomaly detection, where abnormal findings may be rare, diverse, and costly to label. Luo et al. [108] developed a self-supervised contrastive framework for anomaly detection in brain MRI. Their approach contrasts normal and abnormal patches and uses augmented views of the same patch as positives, allowing the model to learn representations sensitive to subtle pathological changes. The study reported improved anomaly detection performance over prior baselines, suggesting that CL can support early detection of neurological abnormalities in settings where annotated anomalies are scarce.

### 4.2. Electronic Health Records

EHRs represent a major application area for CL in medical AI because they capture longitudinal patient trajectories at scale. EHR data are inherently heterogeneous, combining structured variables (e.g., laboratory values, vital signs, diagnosis codes, medications) with unstructured clinical narratives. Compared to imaging, EHR modeling is additionally challenged by irregular sampling, missingness, temporal drift, high dimensionality, and institutional variation in coding and documentation practices. These characteristics often limit the portability of supervised models and increase reliance on large labeled cohorts that may not generalize across healthcare systems. CL offers a promising alternative by enabling representation learning from unlabeled EHR sequences, producing patient embeddings that can transfer effectively across predictive tasks and label-scarce clinical settings.

A common EHR contrastive paradigm constructs multiple views of the same patient record through stochastic perturbations, temporal cropping, modality masking, or aggregation windows, treating these as positive pairs while contrasting against other patients as negatives. Krishnan et al. [109] applied CL to EHR data through a self-supervised framework that generates augmented views of patient histories. By treating augmented versions of the same patient’s record as positives and records from different patients as negatives, the model learns patient representations that preserve temporal and clinical structure. Their experiments showed improved performance compared to standard supervised baselines across multiple tasks, including mortality prediction and heart failure diagnosis, highlighting the role of contrastive pretraining in improving clinical risk stratification under limited labeled data.

Beyond general-purpose representation learning, contrastive learning has also been applied to construct task-ready patient embeddings that support common operational outcomes in healthcare systems. Pick et al. [110] developed a contrastive framework for learning patient-level representations for hospital mortality and length-of-stay prediction. Kerdabadi et al. [111] proposed an ontology-aware temporal contrastive survival framework that learns patient embeddings using temporally distinctive patterns and hardness-aware negatives, demonstrating improved acute kidney injury survival risk prediction. Liu et al. [112] addressed irregular sampling and missingness through a contrastive imputation–prediction network, in which contrastive objectives guide representation learning during data reconstruction, leading to improved in-hospital mortality prediction. Zang and Wang [113] adopted a supervised contrastive framework for longitudinal EHR classification, using label-informed positives to tighten outcome-specific clusters. By explicitly modeling similarity and dissimilarity between patient trajectories, their approach improved downstream risk prediction performance, suggesting that contrastive objectives can better capture latent clinical states compared to purely supervised training on sparse labels.

EHR data are increasingly multimodal, motivating contrastive objectives that align complementary patient information sources rather than learning from each modality independently. Sun et al. [114] developed a CL framework for multimodal EHR integration, aligning structured signals such as laboratory results and vital signs with representations derived from unstructured clinical notes. Positive pairs were constructed by matching structured and unstructured views from the same patient, while negatives were formed using cross-patient mismatches. This alignment improved the quality of patient representations and increased performance in tasks including disease progression prediction and complication risk identification. These results support the growing view that CL is particularly well-suited for EHR settings where clinically meaningful information is distributed across heterogeneous modalities.

Finally, a major barrier to real-world EHR modeling is scale. Health systems produce massive longitudinal datasets that require scalable training strategies and efficient distributed computation. Cai et al. [115] addressed this challenge by proposing a distributed CL framework designed for large-scale EHR repositories. Their work highlights that CL can be extended to big-data healthcare settings and suggests practical pathways for training robust patient representation models across diverse populations.

### 4.3. Genomics and Proteomics

In genomics and proteomics, CL offers substantial advantages for analyzing high-dimensional biomedical data, where labeled outcomes are often limited and experimental noise can be substantial. These domains generate massive quantities of molecular measurements, including DNA variation, gene expression profiles, epigenetic regulation signals, and protein sequences and structures. However, learning clinically meaningful representations remains challenging due to extreme feature dimensionality, sparsity (especially in single-cell assays), batch effects, confounding biological and technical variation, and the need to integrate signals across heterogeneous omics layers. CL is particularly well-suited to this setting because it can learn robust molecular representations by exploiting intrinsic structure, such as similarity across biological replicates, related pathways, shared molecular functions, or paired measurements across modalities, without requiring extensive annotation.

Within genomics, Zhong et al. [116] applied CL to identify disease-associated genetic signals using a multi-scale contrastive learning (MSCL) framework. MSCL was designed to capture genetic interactions across multiple levels of granularity, from local gene patterns to broader pathway-level relationships. By defining positive pairs using sequences from the same genomic regions and negatives from distinct regions, the model learns representations that differentiate healthy from diseased samples and enhances the detection of genetic markers. This multi-scale formulation reflects a key advantage of CL for genomics: contrastive objectives can encode biologically meaningful similarity under complex, non-linear genetic interactions that are difficult to capture with standard supervised pipelines.

A second major direction is multi-omics integration, where the objective is to learn unified molecular patient representations across complementary assays. Liu et al. [117] advanced CL for genomics by developing a framework (MoHeG/GenCL) that aligns heterogeneous omics layers such as genomics, transcriptomics, and epigenomics. Contrastive alignment across modalities enables the model to learn consistent patient-level embeddings that capture regulatory interactions between genes and downstream functional effects. Such unified representations improve interpretability and predictive power for disease susceptibility and precision medicine tasks, particularly in multifactorial diseases where interactions across molecular layers are essential.

Single-cell genomics further amplifies the relevance of CL, since scRNA-seq data are highly sparse, noisy, and sensitive to batch effects, yet rich in unlabeled biological structure. Li et al. [118] proposed a CL framework for scRNA-seq representation learning that constructs positive pairs between cells with similar expression patterns while contrasting against dissimilar cells. This approach improves clustering of cell types and supports the discovery of rare populations, which is critical for understanding tumor microenvironments, immune heterogeneity, and neurodegenerative processes. More broadly, CL offers a natural mechanism for learning invariances to technical noise while retaining biologically meaningful discriminative structure.

In proteomics, CL has been applied to learn protein representations that reflect functional and structural similarity. Bepler and Berger [119] used CL to derive protein sequence representations by forming positive pairs using different conformations or states of the same protein and negatives from unrelated proteins. This formulation improves representation quality for tasks including protein function prediction and interaction modeling, supporting downstream applications in drug discovery and protein engineering.

At the protein interaction level, Zhang et al. [120] developed Pepharmony, a CL approach for predicting protein–protein interactions (PPIs) by integrating both sequence and structural information. Positive pairs were constructed from interacting protein conformations, while negatives corresponded to non-interacting proteins. The resulting embeddings improved PPI prediction accuracy, highlighting that contrastive objectives can capture complex molecular compatibility signals that are essential for understanding disease mechanisms and discovering therapeutic targets.

### 4.4. Multimodal and Cross-Domain Learning

Integrating heterogeneous medical modalities, such as imaging, clinical text, and structured patient data, is a major direction in medical AI, because clinically meaningful decision-making often requires joint reasoning across multiple information sources. CL provides a principled mechanism for multimodal fusion by aligning representations from different modalities into a shared embedding space, enabling label-efficient learning, retrieval, and zero-shot transfer.

Early medical vision–language contrastive models focused on radiology report alignment. Zhang et al. [18] introduced ConVIRT, which learns chest X-ray representations by aligning images with paired radiology reports using a bidirectional contrastive objective. ConVIRT demonstrated substantial label efficiency, requiring only 10% of labeled data relative to an ImageNet-initialized baseline to achieve similar or better performance across four downstream tasks. Building on this paradigm, Huang et al. [65] proposed GLoRIA, which strengthens supervision through both global and local alignment between image regions and report phrases. On MIMIC-CXR, GLoRIA achieved a precision@5 of 69.24% for image-to-text retrieval compared to 66.98% for ConVIRT, and attained CheXpert AUROC scores of 0.926, 0.943, and 0.950 when fine-tuned with 1%, 10%, and 100% labeled data, respectively. Boecking et al. [66] further improved semantic alignment with BioViL by incorporating domain-specific biomedical language pretraining. BioViL achieved zero-shot accuracy, F1, and AUROC of 0.732, 0.665, and 0.831, respectively, and reached a linear-probe AUROC of up to 0.891 on RSNA pneumonia classification, establishing a strong benchmark for biomedical vision–language representation learning.

More recent work has emphasized the importance of temporality and structured clinical priors. Bannur et al. [67] proposed BioViL-T, extending vision–language pretraining with temporal alignment across prior and current chest X-rays. BioViL-T improved progression classification, phrase grounding, and report generation, highlighting the clinical relevance of longitudinal contrastive structure. Similarly, You et al. [68] developed CXR-CLIP, integrating radiologist-defined prompts with both image–label and image–text supervision to improve clinical interpretability and downstream robustness. Collectively, these works suggest that incorporating temporal cues, richer language semantics, and supervised priors improves the clinical validity of contrastive multimodal representations.

Contrastive pretraining has also enabled clinically meaningful zero-shot transfer through large-scale vision–language alignment. Tiu et al. [69] introduced CheXzero, a CLIP-based model trained on unannotated chest X-rays and reports. In a reader study, CheXzero achieved multi-label classification performance statistically indistinguishable from board-certified radiologists on CheXpert, with no significant differences in Matthews correlation coefficient across five evaluated pathologies. To improve robustness under limited or noisy pairings, Wang et al. [70] proposed MedCLIP, which decouples image and text corpora and uses a knowledge-aware matching loss to mitigate false negatives. Using only 20,000 pretraining pairs, MedCLIP achieved a zero-shot accuracy of 44.8%, surpassing GLoRIA (43.3% with 191,000 pairs) and ConVIRT (42.2% with 369,000 pairs) under identical evaluation settings.

Beyond radiology, multimodal CL has advanced computational pathology and strengthened cross-domain generalization. Huang et al. [71] proposed PLIP, a pathology vision–language foundation model trained on OpenPath image–caption pairs. PLIP achieved zero-shot F1 scores between 0.565 and 0.832 across four external datasets, outperforming prior vision–language models that achieved F1 between 0.030 and 0.481. Lu et al. [72] introduced CONCH, trained on over 1.17 million histopathology image–caption pairs, demonstrating state-of-the-art performance across classification, retrieval, captioning, and segmentation tasks. These findings indicate that scaling multimodal contrastive pretraining improves transferability in histopathology, where domain shift across scanners, staining, and cohorts is a pervasive challenge.

Scaling has also expanded to literature-based biomedical corpora, enabling more generalizable biomedical foundation models. Zhang et al. [73] developed BiomedCLIP, pretrained on 15 million image–text pairs from PubMed Central. BiomedCLIP achieved 56% and 77% top-1 and top-5 retrieval accuracy on a 725,000-pair held-out set and demonstrated strong zero- and few-shot performance across radiology and pathology benchmarks, often surpassing domain-specific models such as ConVIRT and GLoRIA. Similarly, Lin et al. [74] proposed PMC-CLIP, pretrained on 1.6 million biomedical figure–caption pairs, improving medical visual question answering and retrieval performance, and highlighting the value of literature-derived multimodal alignment when clinical pairings are scarce.

Recent innovations have moved beyond representation learning to enable fine-grained localization and segmentation. Huang et al. [121] introduced MaCo, which applies masked CL with correlation weighting to chest X-rays and improves both zero-shot and supervised recognition of localized findings. Koleilat et al. [122] combined contrastive vision–language models with the Segment Anything Model to enable text-driven segmentation across ultrasound, MRI, and CT datasets, achieving strong performance without explicit segmentation annotations. Overall, these developments demonstrate the growing versatility of multimodal and cross-domain CL, enabling efficient, interpretable, and transferable medical AI systems that bridge visual and textual clinical evidence.

### 4.5. Time-Series and Physiological Signal Analysis

Medical time-series data, such as ECG, EEG, respiratory signals, and vital signs, present major opportunities for CL because large volumes of unlabeled recordings are routinely collected in clinical practice. However, these signals are also characterized by strong temporal dependencies, noise, missingness, and substantial inter-patient variability. A systematic review by Liu et al. [123] covering 43 studies on self-supervised CL for medical time series reported that most approaches rely on standard augmentations (e.g., scaling, jittering, cropping) and encoder architectures such as 1D CNNs or Transformers. The review further emphasized the need for hierarchical and patient-aware contrastive objectives to better capture long-range dependencies and clinically meaningful temporal consistency.

Diamant et al. [19] introduced Patient Contrastive Learning of Representations (PCLR), which defines positive pairs as ECG recordings from the same patient and negatives as recordings from different patients. Using a dataset of more than 3.2 million 12-lead ECGs, their results showed that linear models trained on PCLR representations achieved an average 51% improvement across downstream tasks, including sex classification, age regression, left ventricular hypertrophy, and atrial fibrillation detection, compared to models trained from scratch. Relative to alternative pretraining strategies, PCLR achieved a 47% average gain on three of four tasks and yielded a 9% improvement over the strongest baseline per task.

Yuan et al. [124] proposed poly-window CL, which samples multiple overlapping temporal windows from each ECG as positive pairs rather than relying on only two augmented views. On the PTB-XL dataset, this approach achieved AUROC 0.891 compared to 0.888 for conventional two-view CL, and an F1 score of 0.680 versus 0.679, while reducing pretraining time by 14.8%. These findings suggest that explicitly modeling intra-record temporal relationships can improve both efficiency and representation quality.

Wang et al. [125] developed COMET, a hierarchical CL framework that organizes data at multiple levels, including observation, sample, trial, and patient, and applies contrastive objectives across these granularities. COMET demonstrated improvements over six baselines across ECG and EEG datasets targeting myocardial infarction, Alzheimer’s disease, and Parkinson’s disease tasks, particularly in low-label settings (10% and 1%). Chen et al. [76] introduced CLOCS (Contrastive Learning of Cardiac Signals across Space, Time, and Patients), which aligns temporal segments and ECG leads to improve robustness under lead variation and temporal drift. These hierarchical and spatiotemporal approaches extend CL beyond simple view augmentation toward multi-level temporal consistency.

Raghu et al. [126] explored multimodal extensions by pretraining contrastive models on physiological time series combined with structured clinical variables such as laboratory values and vital signs. Their results indicated consistent downstream gains compared to baseline pretraining methods, highlighting the value of multimodal temporal alignment for capturing richer clinical context. Guo et al. [127] proposed a Multi-Scale and multimodal contrastive learning network (MBSL) for biomedical signals, leveraging cross-modal contrastive objectives between modalities such as respiration, heart rate, and motion sensors. MBSL reduced mean absolute error by 33.9% for respiration rate prediction, by 13.8% for exercise heart rate estimation, and improved activity recognition accuracy and F1 scores by 1.41% and 1.14%, respectively, compared to state-of-the-art baselines.

To address false negatives arising from batch sampling in large clinical cohorts, Sun et al. [128] proposed a Patient Memory Queue (PMQ) mechanism that maintains a memory bank of intra-patient and inter-patient samples during contrastive pretraining. Across three public ECG datasets and varying label ratios, PMQ outperformed existing contrastive methods in both classification accuracy and robustness to label scarcity. These patient-aware memory designs reflect a broader trend in physiological CL toward objectives that explicitly encode patient identity and long-term temporal consistency.

Overall, CL for physiological time series has progressed from basic augmentation-driven pipelines toward more structured paradigms, including multi-window sampling, hierarchical CL, cross-modal alignment, and patient-aware memory mechanisms. These innovations enable models to better capture temporal dynamics and inter-patient invariances inherent in clinical signals, improving robustness and label efficiency in data-limited healthcare settings. Table 5 summarizes key applications of contrastive learning across different medical domains.

## 5. Challenges, Limitations, and Practical Considerations

Despite strong empirical results, translating contrastive learning into clinically reliable and reproducible medical AI remains challenging. These challenges arise at multiple levels, including pair construction, augmentation design, evaluation methodology, multimodal supervision quality, fairness and privacy risks, optimization stability, and deployment constraints.

### 5.1. Pair Construction and Clinical Semantics

Most contrastive objectives rely on constructing positive and negative pairs, yet defining such pairs in clinical settings is nontrivial. In standard instance discrimination, samples from different patients are treated as negatives by default. However, medical datasets frequently contain semantically similar cases across patients (e.g., shared phenotypes, repeated staging patterns, common radiographic presentations), which can produce false negatives. False negatives attenuate disease-relevant signal by pushing clinically similar cases apart in embedding space and may bias learned representations toward spurious correlates. In addition, patient identity, acquisition protocol, scanner vendor, and site effects may leak into representations if sampling is not controlled. When paired views are constructed within a narrow subset of acquisition conditions, the contrastive objective may prioritize hospital-specific or device-specific features over pathology features. This shortcut learning can yield high in-domain performance while degrading cross-site generalization.

Clinical datasets also exhibit longitudinal structure, with repeated measurements over time. If not accounted for, longitudinal leakage can inflate downstream evaluation: pretraining and fine-tuning may implicitly learn patient-specific signatures rather than clinically generalizable features. Even when patient-level splits are used, subtle overlaps can persist when repeated exams, segments, or derived patches are not tracked carefully. These issues motivate representation-level interpretability and alignment audits to ensure that embedding similarity reflects clinical semantics.

### 5.2. Augmentation and View Design in Medical Data

A key assumption in contrastive learning is that two augmented views of the same instance preserve semantic identity. However, augmentation policies calibrated for natural images do not always transfer to medical data. Geometric transformations, cropping, and intensity perturbations can erase subtle findings, distort anatomical context, or remove small lesions. For example, aggressive cropping can eliminate peripheral abnormalities, while contrast jittering can suppress radiological cues tied to tissue density.

In medical imaging, defining clinically valid invariances requires domain expertise. Certain transformations may be valid for some tasks (e.g., rotation for dermoscopy) but invalid for others (e.g., left-right flips in chest X-rays). Similarly, time-series augmentations such as jittering or permutation can disrupt clinically meaningful rhythms in ECG or EEG. Without medically validated augmentations and consistent reporting of augmentation policy, contrastive pretraining may learn invariances that suppress pathologies or amplify confounders.

### 5.3. Heterogeneity Across Modalities and Tasks

A central challenge for medical CL is heterogeneity across modalities. Imaging, EHR, omics, clinical notes, and physiological signals have fundamentally different noise processes, feature semantics, and temporal structure. A contrastive formulation that works well for radiology may fail in EHR due to sparsity, irregular sampling, and missingness patterns that encode care pathways rather than disease. In genomics and proteomics, the definition of positives and negatives often depends on biological priors, and naive sampling can embed batch effects rather than functional similarity.

Furthermore, clinical tasks span diagnosis, prognosis, retrieval, segmentation, progression monitoring, and phenotyping. Contrastive objectives optimized for global representations may underperform in localization-sensitive tasks (e.g., lesion detection or segmentation), where patch selection and spatial correspondence matter. This mismatch between representation objective and clinical task introduces uncertainty about which CL choices yield clinically meaningful embeddings.

### 5.4. Reproducibility and Reporting Gaps

Reproducibility is constrained by restricted data access, limited release of pretraining corpora, and incomplete reporting. Many papers under-specify key components such as augmentation policy, pairing strategy, preprocessing, tokenizer, and text normalization steps, or hyperparameters (batch size, temperature, queue length). Even when code is released, private clinical datasets and institutional pipelines limit replicability.

CL outcomes are highly sensitive to training details, and small differences in preprocessing can lead to nontrivial changes in downstream performance. This sensitivity makes it difficult to assess whether improvements arise from methodological novelty or from differences in training scale and implementation.

### 5.5. Multimodal Alignment and Weak Supervision Risks

Multimodal contrastive alignment introduces additional concerns. Free-text reports include negations, hedging, and section-specific context that can misalign with image-level findings. A pathology may be present but unmentioned in the report, producing implicit false negatives. Conversely, templated phrases or clinical history can produce matches unrelated to the actual imaging evidence.

Weak supervision mined from reports may propagate label noise if entity linking, uncertainty handling, and negation detection are not explicit. These issues can amplify bias and reduce trustworthiness in downstream clinical interpretation. Additionally, multimodal pretraining can inadvertently learn shortcuts via hospital-specific language patterns, scanner metadata, or demographic correlates embedded in reporting style.

### 5.6. Interpretability and Explainability

Interpretability is central to clinical validity, yet it remains underdeveloped in medical CL. Unlike supervised models, where explanations can be tied directly to task labels, CL pretraining optimizes representation geometry via similarity objectives (e.g., InfoNCE), which can inadvertently encode a mixture of clinically meaningful factors (e.g., anatomy and pathology) and nuisance variables (e.g., site, device, acquisition protocol, reporting style). This is particularly problematic in multi-centre settings, where site effects may dominate embedding structure even when downstream metrics appear strong. Consequently, explainability for CL should emphasize representation-level interpretability, understanding what factors structure the embedding space, rather than only post-hoc explanation of downstream predictions. A practical CL-specific approach is to perform alignment audits to verify whether embedding similarity corresponds to clinical semantics. For example, nearest-neighbor retrieval in embedding space can reveal whether clinically similar cases are clustered together or whether representations separate primarily by hospital/site or scanner vendor. Likewise, embedding visualizations (e.g., UMAP/t-SNE) overlaid with metadata such as site, device, or demographic variables can expose confounding and shortcut learning. Since CL aims to learn invariances defined by augmentations, interpretability can also be operationalized as invariance auditing: representations should remain stable under clinically irrelevant perturbations (e.g., mild intensity changes) while remaining sensitive to clinically meaningful changes.

Caution is warranted when using saliency methods (e.g., Grad-CAM) on downstream classifiers trained atop CL encoders. Such explanations may not reflect what the contrastively learned representation encodes, and may amplify spurious shortcuts (e.g., text markers, laterality cues, portable vs stationary scanner artifacts). We therefore recommend pairing saliency with representation-level audits (retrieval inspection, metadata overlays, invariance tests) and documenting interpretability failure cases, as a minimum standard for clinical transparency.

At minimum, medical CL studies should report: (i) embedding-space retrieval examples with clinician review; (ii) representation visualizations with site/device overlays; (iii) invariance sensitivity tests aligned with clinical plausibility; and (iv) interpretability failure cases highlighting confounding or shortcut reliance.

### 5.7. Fairness, Bias, and Clinical Validity

Fairness concerns remain under-addressed in medical CL. Self-supervised objectives do not eliminate bias. Instead, they may learn and amplify latent dataset biases. If the pretraining distribution is skewed, for example, through over-representation of certain populations or hospital systems, embeddings may transfer poorly to underrepresented groups. Bias may manifest through subgroup performance gaps, miscalibration, or uneven representation quality.

Clinical validity also requires interpretability and subgroup evaluation. Yet many studies rely primarily on global metrics and do not assess whether learned representations preserve clinically relevant features consistently across demographic groups, comorbidity profiles, or rare disease subpopulations.

## 6. Discussion and Future Research Directions

This review shows that CL has evolved from a general-purpose self-supervised paradigm into a versatile representation learning toolkit for medical AI. Across imaging, electronic health records, physiological signals, omics, and multimodal settings, contrastive objectives have consistently demonstrated improved label efficiency and transferable feature learning, especially when pretrained on large-scale unlabeled corpora. Nevertheless, the current evidence base remains uneven across modalities and clinical tasks. Imaging and vision–language learning have benefited from large datasets and standardized benchmarks, whereas time-series, EHR, and omics studies are still characterized by heterogeneous experimental designs, inconsistent evaluation protocols, and limited external validation. As a result, many reported gains remain difficult to compare across studies, and the extent to which improvements persist under realistic clinical deployment conditions is often unclear.

A unifying insight across modalities is that the construction of positive and negative pairs fundamentally determines clinical meaning, downstream robustness, and the risk of shortcut learning. Instance-level positives generated through stochastic augmentations can work well in imaging, but medical settings frequently benefit from pairing strategies grounded in clinical structure, such as patient-level positives derived from repeated exams, longitudinal studies, adjacent slices, multi-view imaging, or multiple recordings from the same patient [76,129]. Conversely, standard negative sampling assumptions often break down in clinical cohorts, where semantically similar patients can appear in the same batch and inadvertently become false negatives. This issue can attenuate disease-relevant signal, degrade calibration, and bias representations toward acquisition artifacts and cohort effects. These risks are amplified in multi-center settings where site-specific protocols, scanner differences, demographic variation, and institutional workflows introduce confounders that may be spuriously predictive, thereby undermining generalization [129,130].

Another consistent finding is that scale improves performance but does not guarantee reliability. Large pretraining corpora may increase representation quality, yet they can simultaneously amplify dataset biases and confounding patterns unless sampling and evaluation explicitly account for distribution shift. In particular, multimodal contrastive alignment has enabled major advances in radiology and pathology by leveraging paired image–report data and CLIP-style retrieval objectives, enabling strong zero-shot and label-free inference [18,66,69]. However, multimodal supervision introduces new failure modes. Radiology reports frequently contain negation, uncertainty, templated phrasing, and contextual descriptions that do not map cleanly to image-level labels, making report–image supervision inherently noisy. Without explicit handling of uncertainty and structured clinical semantics, vision–language alignment can produce misleading associations, propagate label noise, or encourage superficial lexical matching rather than clinically grounded representations.

From a practical clinical AI systems perspective, these findings imply that CL should be treated not merely as a performance optimization tool but as an upstream design decision that shapes downstream validity, safety, and reproducibility. Method choices must be aligned with deployment realities: pairing strategies should reflect clinical semantics rather than convenience; evaluation protocols should match intended clinical use; and robustness under domain shift should be treated as a minimum requirement rather than an optional extension. In addition, clinical adoption depends on more than accuracy. Reporting should increasingly incorporate calibration, uncertainty, and subgroup performance, since miscalibration or uneven error rates across groups can directly translate into inequitable or unsafe decision support [131,132]. Interpretability should also be treated as a core evaluation dimension rather than a future add-on. Even when contrastive pretraining improves end-task scores, the clinical trustworthiness of representations remains limited if models are opaque, brittle under shift, or reliant on shortcuts. Therefore, explanation methods and grounding analyses should be included in model validation pipelines where applicable [133,134,135].

Future research should prioritize directions that maximize clinical impact while closing the methodological gaps identified across the literature. A near-term priority is to establish standardized reporting and benchmarking practices that enable fair comparison across studies, including clear documentation of patient-level split rules, pretraining corpus composition, pairing heuristics, augmentation policies, and key hyperparameters. Similarly, external validation should become routine, with experiments that explicitly examine multi-site generalization, temporal drift, and device/protocol variation [129,136]. Augmentation strategies should be clinically validated, since transformations suitable for natural images may suppress subtle pathology signals or distort medically relevant textures and boundaries. In parallel, the field would benefit from more operational guidance that maps typical medical conditions and data regimes to appropriate contrastive objective families, supporting decision-making such as when negative-free objectives may be preferable, when patient-aware pairing should be emphasized, and when supervised contrastive objectives offer more stable learning in heavily imbalanced multi-label settings.

Beyond near-term standardization, methodological progress should increasingly target known clinical failure modes. Promising directions include debiased objectives, calibrated hard-negative strategies, and supervision schemes that integrate patient groups or clinically meaningful priors. Reducing shortcut learning will likely require approaches that explicitly address confounding, including strategies inspired by invariant learning or causal representation learning [137,138]. For multimodal systems, robust alignment will depend on clinical language understanding that can model negation, uncertainty, and structured report content; entity-level alignment and section-aware objectives may reduce label noise and improve faithfulness. Another important long-term direction concerns privacy-preserving learning, particularly because medical pretraining often requires sensitive data at scale. Federated CL, secure aggregation, and differential privacy mechanisms may provide pathways toward multi-institution representation learning while limiting the risks of memorization and re-identification [139,140]. Finally, the clinical value of contrastive pretraining should be validated not only through retrospective benchmarks but also through prospective and workflow-based studies, including reader-in-the-loop evaluation that measures real endpoints such as reduction in annotation burden, improved diagnostic consistency, or improved time-to-decision [141].

Despite its promise, CL also faces limitations that may not be fully resolvable in the short term. Pair construction and negative sampling remain structurally challenging in medicine because clinical similarity is often latent, labels can be incomplete, and cohort overlap can be high. Domain shift is pervasive and can emerge through unmeasured confounders, evolving documentation practices, changes in acquisition protocols, and institutional workflow differences. While CL can reduce sensitivity to shift, it does not eliminate the need for explicit external validation and careful deployment monitoring. In addition, compute and infrastructure requirements continue to constrain accessibility: large-scale pretraining, comprehensive ablations, and multi-center validation are often feasible only for well-resourced institutions, which can bias the research landscape and limit independent reproducibility. Overall, CL is positioned as a foundational paradigm for medical representation learning and multimodal alignment, but its clinical adoption will depend on moving beyond in-domain accuracy toward robust, reproducible, externally validated, and clinically interpretable evidence under realistic deployment conditions.

## 7. Conclusions

CL has emerged as one of the most influential self-supervised paradigms in medical AI, enabling representation learning from large-scale unlabeled data in settings where annotation is costly, scarce, and heterogeneous. This review synthesized recent progress across medical imaging, electronic health records, physiological time series, omics, and multimodal vision–language systems, and provided an operational taxonomy linking contrastive design choices to clinical data properties. Overall, evidence suggests that contrastive pretraining can improve label efficiency and downstream performance, particularly when positives and augmentations are defined in a clinically meaningful way and evaluation includes external validation.

However, the review also reveals persistent limitations that constrain translation. Reported gains are difficult to compare due to heterogeneous datasets, label taxonomies, and evaluation protocols, and many studies still emphasize in-domain results with limited multi-center validation. Reproducibility remains weak because pretraining corpora, pairing heuristics, augmentation policies, and optimization settings are often incompletely reported, and because many advances rely on proprietary datasets that cannot be audited or replicated. In addition, several limitations may not be resolvable in the short term: false negatives induced by patient similarity, shortcut learning driven by site and acquisition confounders, and noisy supervision in vision–language models arising from negation, uncertainty, and incomplete reporting in free-text clinical notes.

Future progress requires a shift from purely architectural novelty toward clinically grounded evidence. Robust benchmarking with standardized reporting, systematic evaluation under distribution shift, calibration, and subgroup analyses, and privacy-preserving training should become the default expectations. Ultimately, CL is a promising foundation for scalable medical representation learning, but its clinical impact will depend on reproducible methodology and rigorous validation under real-world deployment constraints.

## Figures and Tables

**Table 1:** Summary of representative review papers on contrastive and self-supervised learning, highlighting the scope and coverage of medical applications.

Review	Year	Primary Scope	Medical Coverage	Notes for Positioning in This Paper
Jaiswal et al. [20]	2020	General CL and SSL overview	Limited	Early overview of CL principles and variants.
Gui et al. [21]	2024	Broad SSL survey	Limited	Includes contrastive, generative, and clustering-based SSL methods.
Hu et al. [22]	2024	General CL survey	Indirect	Presents a universal CL framework and component-level advances.
Liu [23]	2021	CL for visual representation	Indirect	Vision-focused synthesis of CL components, limitations, and improvements.
Shurrab et al. [24]	2022	SSL in medical imaging	Imaging only	Focuses on imaging applications with limited clinical data coverage.
Wang et al. [25]	2023	Medical imaging SSL and CL	Imaging only	Emphasizes adaptation of natural image SSL methods to medical imaging.
Huang et al. [26]	2023	Systematic review of SSL for medical imaging	Imaging only	Systematic evidence synthesis and reporting trends for image classification studies.
VanBerlo et al. [27]	2024	Evidence review of SSL pretraining in imaging	Imaging only	Highlights comparisons against supervised baselines and transfer protocols.
**This review**	2025	CL in medical AI	Imaging, EHR, signals, omics, multimodal	Cross modality synthesis with operational taxonomy and evaluation guidance.

**Table 2:** Taxonomy dimensions for medical contrastive learning studies.

Dimension	Definition	Common Categories/Options
Loss family/ objective	The contrastive or self-supervised learning signal is used to shape representations.	InfoNCE/NT-Xent; supervised contrastive; distillation/negative-free; clustering consistency; cross-modal retrieval loss; temporal predictive losses (e.g., CPC).
Pairing strategy (positives)	How positives are constructed, i.e., what the method enforces to be similar.	Augmented views of the same instance; same-class positives; patient-aware positives; longitudinal (same patient across time); cross-modal paired (image–text, ECG–EHR); region–text phrase.
Augmentations/ views	Transformations are used to generate alternative views and define invariances.	Imaging: mild geometry/intensity, modality-specific; signals: masking/jitter/windowing; EHR: time masking/cropping; text: entity-aware processing; multi-view sampling (slices/windows).
Label regime	How labels are used during pretraining (if at all) and during evaluation.	Unsupervised (no labels); weak/self-labels; supervised contrastive; semi-supervised (mix of labeled/unlabeled); label-scarce learning curves.
Evaluation protocol	How the downstream benefit is tested, including whether shift and calibration are considered.	Linear probe; full fine-tuning; few-shot/label fractions; external validation (site/device/time); temporal split; retrieval evaluation (Recall@K); calibration (ECE/Brier) for risk models.
Task family	Clinical endpoint and decision context used for evaluation.	Classification (diagnosis/risk); survival/prognosis; segmentation; detection; retrieval/triage; phenotype discovery/clustering; zero-shot/VLM prompting.

**Table 4:** Common benchmarking protocols and metrics for evaluating medical contrastive learning across modalities.

Protocol	What It Tests	Typical Metrics (Examples)
Linear probe	Representation quality under minimal supervision	AUROC/AUPRC (multi-label); accuracy; macro-F1 (class imbalance); calibration metrics when reported
Full fine-tuning	End-task performance with task-specific adaptation	AUROC/AUPRC; Dice/HD95 for segmentation; patient-level metrics for clinical outcomes
Few-shot and label-scarce	Label efficiency and robustness when annotation is limited	AUROC/F1 vs label fraction; confidence intervals across seeds; sensitivity to class imbalance
External validation and site shift	Generalization across institutions, devices, protocols, and time	Performance drop under shift; subgroup robustness; calibration under shift; domain-wise reporting
Retrieval and alignment (vision–language)	Cross-modal grounding and matching fidelity	Recall@K; median rank; phrase grounding scores; zero-shot AUROC using text prompts

**Table 5:** Summary of applications of contrastive learning in medical AI.

Application Domain	Author(s)	Year	Method	Application
Medical Imaging	Azizi et al. [16]	2021	SimCLR-based pretraining on unlabeled chest X-rays	Learned transferable visual representations for medical image classification and segmentation using unlabeled data.
	Chaitanya et al. [105]	2020	Semi-supervised contrastive framework for MRI	Improved segmentation and classification in MRI with limited labels using augmented positive and negative pairs.
	Ciga et al. [106]	2022	Self-supervised contrastive learning for histopathology	Enhanced cancer detection and tissue differentiation in biopsy samples using augmentation-invariant representations.
	Guo et al. [107]	2023	Multi-scale contrastive loss for cardiac MRI segmentation	Captured both global and local structures, improving accuracy of myocardium and ventricle segmentation.
	Luo et al. [108]	2023	Self-supervised anomaly detection using contrastive loss	Detected abnormal regions in brain MRI scans by distinguishing normal and pathological patches.
Electronic Health Records	Krishnan et al. [109]	2022	Self-supervised contrastive learning on augmented EHR views	Modeled temporal and clinical correlations for mortality and heart failure prediction.
	Pick et al. [110]	2024	Contrastive patient representation learning	Improved prediction of hospital mortality and length-of-stay through patient-level embeddings.
	Sun et al. [114]	2024	Cross-modal contrastive framework for EHR integration	Aligned structured and unstructured EHR data to predict disease progression and complications.
	Cai et al. [115]	2024	Distributed large-scale contrastive learning	Scalable training on large EHR datasets for improved generalization across patient populations.
	Kerdabadi et al. [111]	2023	ontology-aware temporal contrastive survival	Learns temporally distinctive EHR embeddings with hardness-aware negatives for AKI survival risk prediction.
	Liu et al. [112]	2023	Contrastive imputation–prediction network (CL-IPN)	Contrastive-enhanced imputation with patient stratification improves in-hospital mortality prediction under missing/irregular EHRs.
	Zang and Wang [113]	2021	Supervised Contrastive framework using longitudinal EHR	Unified supervised contrastive loss improves EHR classification outcomes.
Genomics and Proteomics	Zhong et al. [116]	2024	Multi-scale contrastive learning (MSCL) for genomics	Identified disease-associated genetic markers by modeling gene and pathway-level interactions.
	Bepler and Berger [119]	2021	Contrastive protein sequence representation learning	Learned structural and functional protein embeddings for improved function prediction and drug discovery.
	Liu et al. [117]	2022	Multi-omics contrastive learning (MoHeG/GenCL)	Integrated genomics, transcriptomics, and epigenomics to predict disease susceptibility and treatment outcomes.
	Li et al. [118]	2024	CellContrast for single-cell RNA sequencing	Enhanced clustering and identification of rare cell types in scRNA-seq data.
	Zhang et al. [120]	2024	Pepharmony: sequence–structure contrastive learning	Predicted protein–protein interactions with improved accuracy using multimodal peptide representations.
Multimodal and Cross-Domain Learning	Zhang et al. [18]	2022	ConVIRT (image–text alignment)	Learned chest X-ray representations by aligning images and radiology reports with bidirectional contrastive loss.
	Huang et al. [65]	2021	GLoRIA (global–local image–text alignment)	Improved retrieval and classification on MIMIC-CXR through local region–phrase alignment.
	Boecking et al. [66]	2022	BioViL (biomedical vision–language model)	Enhanced zero-shot radiology performance using domain-specific text pretraining.
	Bannur et al. [67]	2023	BioViL-T (temporal alignment)	Improved disease progression tracking in chest X-rays via temporal contrastive learning.
	You et al. [68]	2023	CXR-CLIP (prompt-based multimodal CL)	Combined image–label and image–text supervision for robust chest X-ray recognition.
	Tiu et al. [69]	2022	CheXzero (CLIP-style vision–language model)	Achieved radiologist-level zero-shot classification on the CheXpert benchmark.
	Wang et al. [70]	2022	MedCLIP (knowledge-aware matching loss)	Reduced false negatives in radiology by decoupling image–text corpora for efficient pretraining.
	Huang et al. [71]	2023	PLIP (pathology vision–language foundation model)	Achieved state-of-the-art performance in pathology classification and zero-shot transfer.
	Lu et al. [72]	2024	CONCH (large-scale histopathology pretraining)	Trained on 1.17 M image–caption pairs for generalizable pathology retrieval and segmentation.
	Zhang et al. [73]	2023	BiomedCLIP (PubMed multimodal foundation model)	Pretrained on 15 M image–text pairs for broad biomedical zero/few-shot applications.
	Lin et al. [74]	2023	PMC-CLIP (literature-derived pretraining)	Improved biomedical VQA and retrieval from 1.6M figure–caption pairs.
	Huang et al. [121]	2024	MaCo (masked contrastive learning)	Applied to chest X-rays for enhanced zero-shot and localized recognition.
	Koleilat et al. [122]	2024	MedCLIP + SAM (text-driven segmentation)	Enabled multimodal segmentation across ultrasound, MRI, and CT without explicit labels.
Time-Series and Physiological Signals	Liu et al. [123]	2023	Systematic review of contrastive time-series methods	Identified key design trends in self-supervised ECG/EEG contrastive learning.
	Diamant et al. [19]	2022	PCLR (patient-level contrastive learning)	Leveraged same-patient ECGs to improve cardiac disease prediction tasks.
	Yuan et al. [124]	2025	Poly-window contrastive learning	Modeled temporal overlap in ECGs to enhance representation efficiency.
	Wang et al. [125]	2023	COMET (hierarchical contrastive framework)	Applied multi-level contrastive learning for ECG and EEG classification with few labels.
	Chen et al. [76]	2025	CLOCS (spatiotemporal contrastive model)	Improved robustness in cardiac signals under lead and time variation.
	Raghu et al. [126]	2022	Multimodal temporal contrastive pretraining	Integrated physiological signals with lab and vitals data for outcome prediction.
	Guo et al. [127]	2025	MBSL (multi-scale multimodal contrastive learning)	Combined respiration, heart rate, and motion signals for multi-task biomedical inference.
	Sun et al. [128]	2025	PMQ (patient memory queue)	Mitigated false negatives in ECG pretraining by leveraging intra-patient memory banks.

## Data Availability

The original contributions presented in the study are included in the article, further inquiries can be directed to the corresponding authors.

## Abbreviations

The following abbreviations are used in this manuscript:

AI	Artificial Intelligence
AUROC	Area Under the Receiver Operating Characteristic Curve
AUPRC	Area Under the Precision–Recall Curve
BioViL	Biomedical Vision–Language
BioViL-T	Biomedical Vision–Language with Temporal Alignment
BiomedCLIP	Biomedical CLIP
BYOL	Bootstrap Your Own Latent
CheXpert	CheXpert dataset
CL	Contrastive Learning
CLOCS	Contrastive Learning of Cardiac Signals
COMET	Hierarchical contrastive framework
CONCH	Histopathology foundation model
ConVIRT	Contrastive Learning of Visual Representations from Text
CPC	Contrastive Predictive Coding
CT	Computed Tomography
CXR-CLIP	Chest X-Ray CLIP
Dice	Dice coefficient
DINO	Self-Distillation with No Labels
ECG	Electrocardiogram
EEG	Electroencephalogram
EHR(s)	Electronic Health Record(s)
F1	F1 score
GLoRIA	Global–Local image–text alignment in radiology
HD95	95th percentile Hausdorff Distance
ICU	Intensive Care Unit
IID	Independent and Identically Distributed
InfoNCE	Info Noise-Contrastive Estimation (loss)
MaCo	Masked Contrastive Learning
MBSL	Multi-Scale and Multimodal Contrastive Learning
MedCLIP	Medical CLIP
mIoU	mean Intersection over Union
MIMIC-CXR	Medical Information Mart for Intensive Care–Chest X-Ray
MoCo	Momentum Contrast
MRI	Magnetic Resonance Imaging
MSCL	Multi-Scale Contrastive Learning
OOD	Out-of-Distribution
PCLR	Patient Contrastive Learning of Representations
PLIP	Pathology Language–Image Pretraining
PMQ	Patient Memory Queue
PPI(s)	Protein–Protein Interaction(s)
PMC-CLIP	PubMed Central CLIP
RSNA	Radiological Society of North America
SAM	Segment Anything Model
scRNA-seq	single-cell RNA sequencing
SimCLR	Simple Framework for Contrastive Learning of Visual Representations
SSL	Self-Supervised Learning
SupCon	Supervised Contrastive Learning
SwAV	Swapping Assignments between Multiple Views
VQA	Visual Question Answering
1D CNN	One-dimensional Convolutional Neural Network

## Appendix A. Database-Specific Search Strings

We provide the database-specific search strategies used to support reproducibility. Search queries combined contrastive and self-supervised learning terms (e.g., “contrastive learning”, “self-supervised”, SimCLR, MoCo, BYOL) with medical domain keywords (e.g., medical imaging, EHR, genomics, ECG/EEG). Searches were restricted to January 2019–October 2025.

### Appendix A.1. PubMed

**Query:**

( (``contrastive learning’’[Title/Abstract] OR ``self-supervised’’[Title/Abstract] OR ``self supervised’’[Title/Abstract] OR ``representation learning’’[Title/Abstract] OR SimCLR[Title/Abstract] OR MoCo[Title/Abstract] OR BYOL[Title/Abstract] OR ``supervised contrastive’’[Title/Abstract] OR InfoNCE[Title/Abstract] OR CLIP[Title/Abstract]) AND (medical[Title/Abstract] OR healthcare[Title/Abstract] OR clinical[Title/Abstract] OR radiology[Title/Abstract] OR ``chest x-ray’’[Title/Abstract] OR CXR[Title/Abstract] OR MRI[Title/Abstract] OR CT[Title/Abstract] OR pathology[Title/Abstract] OR ``electronic health record’’[Title/Abstract] OR EHR[Title/Abstract] OR ``clinical notes’’[Title/Abstract] OR ECG[Title/Abstract] OR EEG[Title/Abstract] OR ``physiological signals’’[Title/Abstract] OR genomics[Title/Abstract] OR proteomics[Title/Abstract] OR ``single-cell’’[Title/Abstract] OR ``multi-omics’’[Title/Abstract]) )

**Filters:** Publication dates: 1 January 2019–31 October 2025; Article types: journal articles and conference papers where applicable; Language: English.

### Appendix A.2. IEEE Xplore

**Query (All Metadata):**

(``contrastive learning’’ OR ``self-supervised’’ OR ``self supervised’’ OR ``representation learning’’ OR SimCLR OR MoCo OR BYOL OR ``supervised contrastive’’ OR InfoNCE OR CLIP) AND (medical OR healthcare OR clinical OR radiology OR ``chest x-ray’’ OR CXR OR MRI OR CT OR pathology OR ``electronic health record’’ OR EHR OR ``clinical notes’’ OR ECG OR EEG OR ``physiological signals’’ OR genomics OR proteomics OR ``single-cell’’ OR ``multi-omics’’)

**Filters:** Years: 2019–2025; Content type: Journals and Conferences; Fields searched: All Metadata.

### Appendix A.3. ACM Digital Library

**Query:**

(``contrastive learning’’ OR ``self-supervised’’ OR ``representation learning’’ OR SimCLR OR MoCo OR BYOL OR ``supervised contrastive’’ OR InfoNCE OR CLIP) AND (medical OR healthcare OR clinical OR radiology OR ``chest x-ray’’ OR MRI OR CT OR pathology OR ``electronic health record’’ OR EHR OR ``clinical notes’’ OR ECG OR EEG OR ``physiological signals’’ OR genomics OR proteomics OR ``single-cell’’ OR ``multi-omics’’)

**Filters:** Publication years: 2019–2025.

### Appendix A.4. Web of Science

**Query (Topic search, TS):**

TS=( (``contrastive learning’’ OR ``self-supervised’’ OR ``self supervised’’ OR ``representation learning’’ OR SimCLR OR MoCo OR BYOL OR ``supervised contrastive’’ OR InfoNCE OR CLIP) AND (medical OR healthcare OR clinical OR radiology OR ``chest x-ray’’ OR CXR OR MRI OR CT OR pathology OR ``electronic health record’’ OR EHR OR ``clinical notes’’ OR ECG OR EEG OR ``physiological signals’’ OR genomics OR proteomics OR ``single-cell’’ OR ``multi-omics’’) )

**Filters:** Timespan: 2019–2025; Document types: Article, Proceedings Paper; Language: English.

### Appendix A.5. Scopus

**Query (TITLE-ABS-KEY):**

TITLE-ABS-KEY( (``contrastive learning’’ OR ``self-supervised’’ OR ``self supervised’’ OR ``representation learning’’ OR SimCLR OR MoCo OR BYOL OR ``supervised contrastive’’ OR InfoNCE OR CLIP) AND (medical OR healthcare OR clinical OR radiology OR ``chest x-ray’’ OR CXR OR MRI OR CT OR pathology OR ``electronic health record’’ OR EHR OR ``clinical notes’’ OR ECG OR EEG OR ``physiological signals’’ OR genomics OR proteomics OR ``single-cell’’ OR ``multi-omics’’) )

**Filters:** Year: 2019–2025; Document type: Article, Conference Paper; Language: English.

### Appendix A.6. arXiv

**Query:**

(``contrastive learning’’ OR ``self-supervised’’ OR ``representation learning’’ OR SimCLR OR MoCo OR BYOL OR ``supervised contrastive’’ OR InfoNCE OR CLIP) AND (medical OR healthcare OR clinical OR radiology OR ``chest x-ray’’ OR MRI OR CT OR pathology OR ``electronic health record’’ OR EHR OR ``clinical notes’’ OR ECG OR EEG OR ``physiological signals’’ OR genomics OR proteomics OR ``single-cell’’ OR ``multi-omics’’)

**Filters:** Years: 2019–2025; Categories considered: cs.LG, cs.CV, cs.AI, eess.IV, q-bio.QM.

## Appendix B. Reporting Checklist

**Table A1:** Lightweight methodological and reporting checklist used to characterize included medical contrastive learning studies. Items are recorded as Yes/No/Unclear. The checklist is descriptive and was not used to exclude studies.

Checklist Item	Rationale for Medical CL
Patient-level split reported (when applicable)	Reduces leakage from repeated exams/segments across train and test.
External validation (cross-site/device/cohort)	Supports generalization beyond in-domain evaluation.
Pretraining corpus described (size, source, pairing)	Enables reproducibility and interpretation of representation quality.
Pairing strategy specified (what defines positives/negatives)	Central determinant of clinical semantics and false-negative risk.
Augmentation policy specified and clinically justified	Prevents invariances that erase subtle pathology or amplify artifacts.
Evaluation regime clearly stated (linear probe, fine-tune, few-shot, zero-shot)	Needed for fair comparison across studies.
Primary metric(s) justified for task (e.g., AUROC vs F1)	Metrics have different clinical meanings under imbalance and thresholding.
Baselines appropriate (supervised, SSL, ImageNet, CL variants)	Prevents inflated claims due to weak comparisons.
Code/model released or sufficient implementation detail provided	Enables replication and reuse.
Reproducibility constraints discussed (private data, restricted access)	Helps interpret evidence strength and limitations.

## Appendix C. Included Studies Summary Table

This section presents a structured taxonomy of the included contrastive learning studies (n = 38). For each study, we summarize (i) biomedical modality and dataset(s), (ii) clinical prediction or analysis task, (iii) contrastive learning formulation and pairing strategy, (iv) label regime and evaluation protocol, and (v) the primary metric used for reporting performance. This taxonomy is intended to improve reproducibility, support cross-modality synthesis, and clarify how contrastive design choices map to clinical data characteristics.

**Table A2:** Summary of included studies (n = 38): modality, dataset, task, contrastive formulation, label regime, evaluation protocol, and key metric.

Modality	Study	Year	Dataset(s)	Task	Contrastive Formulation	Label Regime	Eval Protocol	Key Metric
CXR	Azizi et al. [16]	2021	Large-scale chest X-rays (multi-source CXR)	Classification/transfer	Image–image CL (SimCLR-style): augmented views positives; others negatives	SSL pretrain; supervised transfer	IID and external transfer evaluation	AUROC/Acc
MRI	Chaitanya et al. [105]	2020	Cardiac/brain MRI segmentation benchmarks	Segmentation (limited labels)	Semi-supervised CL: consistency + image-view positives/negatives	SS (few labels + unlabeled)	CV or held-out split	Dice/mIoU
Histopathology	Ciga et al. [106]	2022	WSI patch datasets (public pathology cohorts)	Cancer/tissue classification	Patch–patch SSL CL (augmentation-invariant representations)	SSL pretrain; supervised fine-tune	Patient-level IID split (where applicable)	AUROC/F1
Cardiac MRI	Guo et al. [107]	2023	Cardiac MRI segmentation dataset(s)	Segmentation (myocardium/ventricles)	Multi-scale CL loss (global/local scale alignment)	Supervised + auxiliary CL	Train/val/test or CV	Dice
Brain MRI	Luo et al. [108]	2023	Brain MRI anomaly datasets	Anomaly detection/localization	Patch-level CL (normality representation learning)	SSL/weak supervision	IID split; lesion localization eval	AUROC/ AUPRC
EHR	Krishnan et al. [109]	2022	ICU/hospital EHR cohort(s)	Mortality/HF prediction	Patient view–view CL: time-window crops, code dropout/shuffle	SSL + supervised downstream	IID and/or temporal split	AUROC
EHR	Pick et al. [110]	2024	Hospital/ICU EHR cohort(s)	Mortality + length-of-stay	Patient embedding CL (same-patient/clinical-views positives)	SSL/weak sup + supervised eval	IID or temporal split	AUROC/ MAE
EHR (codes + notes)	Sun et al. [114]	2024	Paired structured EHR + clinical notes	Progression/ complications	Cross-modal CL (align code-based and note-based embeddings)	Weak supervision (paired modalities)	IID; optional cross-site test	AUROC
EHR (large-scale)	Cai et al. [115]	2024	Large EHR warehouse(s)	Generalizable patient modeling	Distributed CL pretraining (large memory bank/shards)	SSL pretrain; supervised fine-tune	Cross-population/cross-site when available	AUROC
EHR survival	Kerdabadi et al. [111]	2023	Large longitudinal EHR cohort for AKI risk forecasting	Survival risk prediction (AKI)	Temporal distinctiveness CL: hardness-aware negative sampling; time-aware pairs	Supervised survival + CL auxiliary loss	Patient-level split; temporal evaluation	C-index/ AUROC
EHR (missing/irregular)	Liu et al. [112]	2023	Two real-world EHR datasets (ICU/hospital cohorts)	In-hospital mortality prediction (with imputation)	CL-imputation–prediction network: patient stratification + contrastive repr. learning	Supervised prediction + CL auxiliary	IID split; patient-level where applicable	AUROC
EHR	Zang and Wang [113]	2021	Longitudinal EHR cohorts (risk prediction benchmarks)	Mortality + phenotyping (multi-label)	Supervised CL loss: contrastive cross-entropy + supervised CL regularizer	Supervised	IID split	AUROC/ micro-F1
Genomics	Zhong et al. [116]	2024	Bulk genomics datasets (expression/pathways)	Biomarker discovery/prediction	Multi-scale CL (gene-level + pathway-level alignment)	Supervised + CL regularization or SSL + FT	IID; CV common	AUROC/F1
Proteins	Bepler and Berger [119]	2021	Protein sequence corpora (+ structures)	Function/structure prediction	Contrastive protein sequence representations	SSL pretrain; supervised downstream	IID; family holdout possible	Acc/AUROC
Multi-omics	Liu et al. [117]	2022	Multi-omics cohorts (paired omics)	Disease susceptibility/outcome prediction	Cross-omics CL: align patient embeddings across modalities	Weak sup (paired omics) + supervised task	IID; CV	AUROC
scRNA-seq	Li et al. [118]	2024	Multiple scRNA-seq datasets	Clustering/rare cell discovery	Cell–cell CL with augmented count views; batch robustness	SSL	Cross-dataset/batch-aware evaluation	ARI/NMI
Seq + structure	Zhang et al. [120]	2024	Paired peptide sequence–structure datasets	PPI prediction	Sequence–structure CL alignment	Weak sup (paired modalities) + supervised	IID + cold-start split	AUROC
CXR + report	Zhang et al. [18]	2022	Paired CXR–report corpora (e.g., MIMIC-CXR)	CXR repr. learning; classification/retrieval	Image–text CL (CLIP-style)	Weak sup (paired)	IID; external transfer	AUROC/ Recall@K
CXR + report	Huang et al. [65]	2021	MIMIC-CXR	Classification/retrieval/ grounding	Global–local image–text CL (region–phrase + global)	Weak sup (paired)	IID; external optional	AUROC/ Recall@K
CXR + report	Boecking et al. [66]	2022	Large CXR-report corpora	ZS/FS radiology benchmarks	BioViL vision–language CL	Weak sup (paired) + ZS eval	IID; external	AUROC
CXR (temporal)	Bannur et al. [67]	2023	MIMIC-CXR (longitudinal pairs)	Disease progression tracking	Temporal CL across patient studies/time	Weak sup (paired + temporal IDs)	Patient split; temporal test	AUROC
CXR + prompt	You et al. [68]	2023	CheXpert/MIMIC-CXR	Prompt-based recognition	Prompt/label-text alignment CL	Weak sup + supervised finetune	IID; external	AUROC
CXR (ZS)	Tiu et al. [69]	2022	Pretrain paired CXR–report; eval CheXpert	Zero-shot multi-label classification	CLIP-style image–text CL; prompt inference	Weak sup + ZS eval	External benchmark	AUROC
Radiology VLP	Wang et al. [70]	2022	Paired + unpaired image/text corpora	Radiology classification robustness	Decoupled/knowledge-aware CL matching	Weak supervision	IID; external validation	AUROC
Pathology VLP	Huang et al. [71]	2023	Pathology image–text corpora	Zero-shot pathology transfer	CLIP-style PLIP	Weak sup (paired)	Cross-dataset transfer	AUROC
Histo VLP	Lu et al. [72]	2024	1.17 M histopathology image–caption pairs	Retrieval/segmentation transfer	Large-scale image–text CL	Weak sup	Multi-task transfer evaluation	AUROC/ Dice
Biomedical VLP	Zhang et al. [73]	2023	PubMed-scale biomedical image–text	Zero/few-shot across tasks	CLIP-style biomedical CL	Weak sup	Benchmark suite	AUROC/ Recall@K
PMC VLP	Lin et al. [74]	2023	1.6 M PMC figure–caption pairs	VQA/retrieval	Figure–caption image–text CL	Weak sup	Benchmark evaluation	VQA Acc/ Recall@K
CXR masked CL	Huang et al. [121]	2024	MIMIC-CXR/CheXpert	ZS + localization	Masked CL + image–text alignment	Weak sup	IID + external evaluation	AUROC
Segmentation (multi)	Koleilat et al. [122]	2024	Ultrasound/MRI/CT segmentation sets	Text-driven segmentation	MedCLIP + SAM; text prompts guide masks	Weak sup (prompts)	Multi-dataset transfer	Dice/mIoU
EHR (structured)	Liu et al. [112]	2023	MIMIC-III; eICU	Clinical time-series imputation + in-hospital mortality prediction	Imputation: unsupervised CL (patient vs augmented view)	Hybrid: SSL (imputation) + supervised (mortality labels)	Random split 70/15/15 train/val/test; repeated 10 runs	Imputation: MAE/MRE; Prediction: AUROC/AUPRC
ECG	Diamant et al. [19]	2022	Repeated ECG per patient cohorts	Cardiac disease prediction	Patient-level CL (same-patient positives)	SSL + supervised downstream	Patient split	AUROC
ECG	Yuan et al. [124]	2025	ECG recordings (windowed)	Classification repr. learning	Poly-window CL (overlap-aware positives)	SSL	IID/patient split	AUROC
ECG/EEG	Wang et al. [125]	2023	ECG and/or EEG datasets	Few-label classification	Hierarchical CL (instance/segment/patient)	SSL/SS + supervised FT	Few-label protocol	AUROC/F1
ECG	Chen et al. [76]	2025	Multi-lead ECG datasets	Robust classification (lead/time shift)	Spatiotemporal CL (lead-wise + time-wise invariance)	SSL	Patient split; robustness tests	AUROC
Physio + labs	Raghu et al. [126]	2022	Multimodal ICU time-series	Outcome prediction	Multimodal temporal CL (align modalities/time contexts)	Weak sup (paired)	Temporal or IID split	AUROC
Wearables	Guo et al. [127]	2025	Respiration/HR/ motion datasets	Multi-task inference	Multi-scale multimodal CL	SSL + supervised tasks	IID/subject split	AUROC/F1
ECG	Sun et al. [128]	2025	Repeated ECG per patient datasets	Reduce false negatives in CL pretraining	Patient memory queue (intra-patient positives)	SSL + supervised downstream	Patient split	AUROC

## References

[1] Parvin N., Joo S.W., Jung J.H., Mandal T.K. (2025). Multimodal AI in Biomedicine: Pioneering the Future of Biomaterials, Diagnostics, and Personalized Healthcare. Nanomaterials.

[2] Nazir A., Hussain A., Singh M., Assad A. (2025). Deep learning in medicine: Advancing healthcare with intelligent solutions and the future of holography imaging in early diagnosis. Multimed. Tools Appl..

[3] Mienye I.D., Swart T.G., Obaido G., Jordan M., Ilono P. (2025). Deep convolutional neural networks in medical image analysis: A review. Information.

[4] Mienye I.D., Jere N., Obaido G., Ogunruku O.O., Esenogho E., Modisane C. (2025). Large language models: An overview of foundational architectures, recent trends, and a new taxonomy. Discov. Appl. Sci..

[5] Nichyporuk B., Cardinell J., Szeto J., Mehta R., Falet J.P.R., Arnold D.L., Tsaftaris S.A., Arbel T. (2022). Rethinking generalization: The impact of annotation style on medical image segmentation. arXiv.

[6] Daneshjou R., Yuksekgonul M., Cai Z.R., Novoa R., Zou J.Y. (2022). Skincon: A skin disease dataset densely annotated by domain experts for fine-grained debugging and analysis. Adv. Neural Inf. Process. Syst..

[7] Krenzer A., Makowski K., Hekalo A., Fitting D., Troya J., Zoller W.G., Hann A., Puppe F. (2022). Fast machine learning annotation in the medical domain: A semi-automated video annotation tool for gastroenterologists. Biomed. Eng. Online.

[8] Chen H., Gouin-Vallerand C., Bouchard K., Gaboury S., Couture M., Bier N., Giroux S. (2024). Contrastive Self-Supervised Learning for Sensor-Based Human Activity Recognition: A Review. IEEE Access.

[9] Liu S., Zhao L., Chen D., Song Z. (2024). Contrastive learning for image complexity representation. arXiv.

[10] Ren X., Wei W., Xia L., Huang C. (2025). A comprehensive survey on self-supervised learning for recommendation. ACM Comput. Surv..

[11] Prince J.S., Alvarez G.A., Konkle T. (2024). Contrastive learning explains the emergence and function of visual category-selective regions. Sci. Adv..

[12] Albelwi S. (2022). Survey on self-supervised learning: Auxiliary pretext tasks and contrastive learning methods in imaging. Entropy.

[13] Khan A., Asmatullah L., Malik A., Khan S., Asif H. (2025). A Survey on Self-supervised Contrastive Learning for Multimodal Text-Image Analysis. arXiv.

[14] Zeng D., Wu Y., Hu X., Xu X., Shi Y. (2024). Contrastive learning with synthetic positives. Proceedings of the European Conference on Computer Vision.

[15] Xu Z., Dai Y., Liu F., Wu B., Chen W., Shi L. (2024). Swin MoCo: Improving parotid gland MRI segmentation using contrastive learning. Med. Phys..

[16] Azizi S., Mustafa B., Ryan F., Beaver Z., Freyberg J., Deaton J., Loh A., Karthikesalingam A., Kornblith S., Chen T. Big self-supervised models advance medical image classification. Proceedings of the IEEE/CVF International Conference on Computer Vision.

[17] Sowrirajan H., Yang J., Ng A.Y., Rajpurkar P. (2021). Moco pretraining improves representation and transferability of chest x-ray models. Proceedings of the Medical Imaging with Deep Learning.

[18] Zhang Y., Jiang H., Miura Y., Manning C.D., Langlotz C.P. (2022). Contrastive learning of medical visual representations from paired images and text. Proceedings of the Machine Learning for Healthcare Conference.

[19] Diamant N., Reinertsen E., Song S., Aguirre A.D., Stultz C.M., Batra P. (2022). Patient contrastive learning: A performant, expressive, and practical approach to electrocardiogram modeling. PLoS Comput. Biol..

[20] Jaiswal A., Babu A.R., Zadeh M.Z., Banerjee D., Makedon F. (2020). A survey on contrastive self-supervised learning. Technologies.

[21] Gui J., Chen T., Zhang J., Cao Q., Sun Z., Luo H., Tao D. (2024). A Survey on Self-supervised Learning: Algorithms, Applications, and Future Trends. IEEE Trans. Pattern Anal. Mach. Intell..

[22] Hu H., Wang X., Zhang Y., Chen Q., Guan Q. (2024). A comprehensive survey on contrastive learning. Neurocomputing.

[23] Liu R. (2021). Understand and improve contrastive learning methods for visual representation: A review. arXiv.

[24] Shurrab S., Duwairi R. (2022). Self-supervised learning methods and applications in medical imaging analysis: A survey. PeerJ Comput. Sci..

[25] Wang W.C., Ahn E., Feng D., Kim J. (2023). A review of predictive and contrastive self-supervised learning for medical images. Mach. Intell. Res..

[26] Huang S.C., Pareek A., Jensen M., Lungren M.P., Yeung S., Chaudhari A.S. (2023). Self-supervised learning for medical image classification: A systematic review and implementation guidelines. NPJ Digit. Med..

[27] VanBerlo B., Hoey J., Wong A. (2024). A survey of the impact of self-supervised pretraining for diagnostic tasks in medical X-ray, CT, MRI, and ultrasound. BMC Med. Imaging.

[28] Yeh C.H., Hong C.Y., Hsu Y.C., Liu T.L., Chen Y., LeCun Y. (2022). Decoupled contrastive learning. Proceedings of the European Conference on Computer Vision.

[29] Khosla P., Teterwak P., Wang C., Sarna A., Tian Y., Isola P., Maschinot A., Liu C., Krishnan D. (2020). Supervised contrastive learning. Adv. Neural Inf. Process. Syst..

[30] Tian Y., Sun C., Poole B., Krishnan D., Schmid C., Isola P. (2020). What makes for good views for contrastive learning?. Adv. Neural Inf. Process. Syst..

[31] Kalantidis Y., Sariyildiz M.B., Pion N., Weinzaepfel P., Larlus D. (2020). Hard negative mixing for contrastive learning. Adv. Neural Inf. Process. Syst..

[32] Wu J., Chen J., Wu J., Shi W., Wang X., He X. (2024). Understanding contrastive learning via distributionally robust optimization. Adv. Neural Inf. Process. Syst..

[33] Le-Khac P.H., Healy G., Smeaton A.F. (2020). Contrastive representation learning: A framework and review. IEEE Access.

[34] Falcon W., Cho K. (2020). A framework for contrastive self-supervised learning and designing a new approach. arXiv.

[35] Peng X., Wang K., Zhu Z., Wang M., You Y. Crafting better contrastive views for siamese representation learning. Proceedings of the IEEE/CVF Conference on Computer Vision and Pattern Recognition.

[36] Kim B., Ye J.C. (2022). Energy-based contrastive learning of visual representations. Adv. Neural Inf. Process. Syst..

[37] Wu L., Zhuang J., Chen H. Voco: A simple-yet-effective volume contrastive learning framework for 3d medical image analysis. Proceedings of the IEEE/CVF Conference on Computer Vision and Pattern Recognition.

[38] Tang C., Zeng X., Zhou L., Zhou Q., Wang P., Wu X., Ren H., Zhou J., Wang Y. (2024). Semi-supervised medical image segmentation via hard positives oriented contrastive learning. Pattern Recognit..

[39] Kundu R. (2022). The Beginner’s Guide to Contrastive Learning. https://www.v7labs.com/blog/contrastive-learning-guide.

[40] Zhang C., Zhang K., Pham T.X., Niu A., Qiao Z., Yoo C.D., Kweon I.S. Dual temperature helps contrastive learning without many negative samples: Towards understanding and simplifying moco. Proceedings of the IEEE/CVF Conference on Computer Vision and Pattern Recognition.

[41] Hoffmann D.T., Behrmann N., Gall J., Brox T., Noroozi M. Ranking info noise contrastive estimation: Boosting contrastive learning via ranked positives. Proceedings of the AAAI Conference on Artificial Intelligence.

[42] Xu L., Xie H., Li Z., Wang F.L., Wang W., Li Q. (2023). Contrastive learning models for sentence representations. ACM Trans. Intell. Syst. Technol..

[43] Chen T., Kornblith S., Norouzi M., Hinton G. (2020). A simple framework for contrastive learning of visual representations. Proceedings of the International Conference on Machine Learning.

[44] Zhang H., Cao Y. (2024). Understanding the benefits of simclr pre-training in two-layer convolutional neural networks. arXiv.

[45] Bunyang S., Thedwichienchai N., Pintong K., Lael N., Kunaborimas W., Boonrat P., Siriborvornratanakul T. (2023). Self-supervised learning advanced plant disease image classification with SimCLR. Adv. Comput. Intell..

[46] Fırıldak K., Çelik G., Talu M.F. (2024). SimCLR-based Self-Supervised Learning Approach for Limited Brain MRI and Unlabeled Images. Bitlis Eren Üniv. Fen Bilim. Derg..

[47] Chen X., Fan H., Girshick R., He K. (2020). Improved baselines with momentum contrastive learning. arXiv.

[48] Li Y., Liu Q., Zhou L., Zhao W., Tian Y., Zhang W. (2023). Improved contrastive learning with MoCo framework. Proceedings of the 2023 3rd International Conference on Consumer Electronics and Computer Engineering (ICCECE).

[49] He Y., Wang X., Shi T. (2024). Ddpm-moco: Advancing industrial surface defect generation and detection with generative and contrastive learning. Proceedings of the International Joint Conference on Artificial Intelligence.

[50] Xie E., Ding J., Wang W., Zhan X., Xu H., Sun P., Li Z., Luo P. Detco: Unsupervised contrastive learning for object detection. Proceedings of the IEEE/CVF International Conference on Computer Vision.

[51] Xiao T., Wang X., Efros A.A., Darrell T. (2020). What should not be contrastive in contrastive learning. arXiv.

[52] Grill J.B., Strub F., Altché F., Tallec C., Richemond P., Buchatskaya E., Doersch C., Avila Pires B., Guo Z., Gheshlaghi Azar M. (2020). Bootstrap your own latent-a new approach to self-supervised learning. Adv. Neural Inf. Process. Syst..

[53] Deng Z., Man J., Song Z., Yang G. (2024). A Few-Shot Anomaly Detection Method Based on BYOL Contrastive Learning Framework. Proceedings of the 2024 4th International Conference on Robotics, Automation and Intelligent Control (ICRAIC).

[54] Richemond P.H., Grill J.B., Altché F., Tallec C., Strub F., Brock A., Smith S., De S., Pascanu R., Piot B. (2020). Byol works even without batch statistics. arXiv.

[55] Caron M., Touvron H., Misra I., Jégou H., Mairal J., Bojanowski P., Joulin A. Emerging properties in self-supervised vision transformers. Proceedings of the IEEE/CVF International Conference on Computer Vision.

[56] Takanami K., Takahashi T., Sakata A. (2025). The effect of optimal self-distillation in noisy gaussian mixture model. arXiv.

[57] Wang Q., Mao Z., Gao J., Zhang Y. (2024). Document-level relation extraction with progressive self-distillation. ACM Trans. Inf. Syst..

[58] Tong S., Xia Z., Alahi A., He X., Shi Y. GeoDistill: Geometry-Guided Self-Distillation for Weakly Supervised Cross-View Localization. Proceedings of the IEEE/CVF International Conference on Computer Vision.

[59] Chen X., He K. Exploring simple siamese representation learning. Proceedings of the IEEE/CVF Conference on Computer Vision and Pattern Recognition.

[60] Zhang C., Zhang K., Zhang C., Pham T.X., Yoo C.D., Kweon I.S. (2022). How does simsiam avoid collapse without negative samples? A unified understanding with self-supervised contrastive learning. arXiv.

[61] Lu Y., Jha A., Deng R., Huo Y. (2022). Contrastive learning meets transfer learning: A case study in medical image analysis. Proceedings of the Medical Imaging 2022: Computer-Aided Diagnosis.

[62] Khorram S., Kim J., Tripathi A., Lu H., Zhang Q., Sak H. (2022). Contrastive siamese network for semi-supervised speech recognition. Proceedings of the ICASSP 2022—2022 IEEE International Conference on Acoustics, Speech and Signal Processing (ICASSP).

[63] Caron M., Misra I., Mairal J., Goyal P., Bojanowski P., Joulin A. (2020). Unsupervised learning of visual features by contrasting cluster assignments. Adv. Neural Inf. Process. Syst..

[64] Radford A., Kim J.W., Hallacy C., Ramesh A., Goh G., Agarwal S., Sastry G., Askell A., Mishkin P., Clark J. (2021). Learning transferable visual models from natural language supervision. Proceedings of the International Conference on Machine Learning.

[65] Huang S.C., Shen L., Lungren M.P., Yeung S. Gloria: A multimodal global-local representation learning framework for label-efficient medical image recognition. Proceedings of the IEEE/CVF International Conference on Computer Vision.

[66] Boecking B., Usuyama N., Bannur S., Castro D.C., Schwaighofer A., Hyland S., Wetscherek M., Naumann T., Nori A., Alvarez-Valle J. (2022). Making the most of text semantics to improve biomedical vision–language processing. Proceedings of the European Conference on Computer Vision.

[67] Bannur S., Hyland S., Liu Q., Perez-Garcia F., Ilse M., Castro D.C., Boecking B., Sharma H., Bouzid K., Thieme A. Learning to exploit temporal structure for biomedical vision-language processing. Proceedings of the IEEE/CVF Conference on Computer Vision and Pattern Recognition.

[68] You K., Gu J., Ham J., Park B., Kim J., Hong E.K., Baek W., Roh B. (2023). Cxr-clip: Toward large scale chest x-ray language-image pre-training. Proceedings of the International Conference on Medical Image Computing and Computer-Assisted Intervention.

[69] Tiu E., Talius E., Patel P., Langlotz C.P., Ng A.Y., Rajpurkar P. (2022). Expert-level detection of pathologies from unannotated chest X-ray images via self-supervised learning. Nat. Biomed. Eng..

[70] Wang Z., Wu Z., Agarwal D., Sun J. Medclip: Contrastive learning from unpaired medical images and text. Proceedings of the Conference on Empirical Methods in Natural Language Processing. Conference on Empirical Methods in Natural Language Processing.

[71] Huang Z., Bianchi F., Yuksekgonul M., Montine T.J., Zou J. (2023). A visual–language foundation model for pathology image analysis using medical twitter. Nat. Med..

[72] Lu M.Y., Chen B., Williamson D.F., Chen R.J., Liang I., Ding T., Jaume G., Odintsov I., Le L.P., Gerber G. (2024). A visual-language foundation model for computational pathology. Nat. Med..

[73] Zhang S., Xu Y., Usuyama N., Xu H., Bagga J., Tinn R., Preston S., Rao R., Wei M., Valluri N. (2023). Biomedclip: A multimodal biomedical foundation model pretrained from fifteen million scientific image-text pairs. arXiv.

[74] Lin W., Zhao Z., Zhang X., Wu C., Zhang Y., Wang Y., Xie W. (2023). Pmc-clip: Contrastive language-image pre-training using biomedical documents. Proceedings of the International Conference on Medical Image Computing and Computer-Assisted Intervention.

[75] van den Oord A., Li Y., Vinyals O. (2018). Representation learning with contrastive predictive coding. arXiv.

[76] Chen W., Wang H., Zhang L., Zhang M. (2025). Temporal and spatial self supervised learning methods for electrocardiograms. Sci. Rep..

[77] Chuang C.Y., Robinson J., Lin Y.C., Torralba A., Jegelka S. (2020). Debiased contrastive learning. Adv. Neural Inf. Process. Syst..

[78] Robinson J., Sun L., Yu K., Batmanghelich K., Jegelka S., Sra S. (2021). Can contrastive learning avoid shortcut solutions?. Adv. Neural Inf. Process. Syst..

[79] Jang T., Wang X. Difficulty-based sampling for debiased contrastive representation learning. Proceedings of the IEEE/CVF Conference on Computer Vision and Pattern Recognition.

[80] Biswas D., Tešić J. (2024). Unsupervised domain adaptation with debiased contrastive learning and support-set guided pseudolabeling for remote sensing images. IEEE J. Sel. Top. Appl. Earth Obs. Remote Sens..

[81] Agarwal A., Banerjee T., Romine W.L., Cajita M. (2024). Debias-clr: A contrastive learning based debiasing method for algorithmic fairness in healthcare applications. Proceedings of the 2024 IEEE International Conference on Big Data (BigData).

[82] Yun B., Zhao S., Li Q., Kot A., Wang Y. (2025). Debiasing Medical Knowledge for Prompting Universal Model in CT Image Segmentation. IEEE Trans. Med. Imaging.

[83] Tang P., Ouyang C., Liu Y. (2023). Debiasing medication recommendation with counterfactual analysis. Proceedings of the International Conference on Neural Information Processing.

[84] Johnson A.E.W., Pollard T.J., Berkowitz S.J., Greenbaum N.R., Lungren M.P., Deng C.Y., Mark R.G., Horng S. (2019). MIMIC-CXR: A large publicly available database of labeled chest radiographs. arXiv.

[85] Irvin J., Rajpurkar P., Ko M., Yu Y., Ciurea-Ilinca M., Chute C., Marklund H., Haghgoo B., Ball R.L., Shpanskaya K. (2019). CheXpert: A Large Chest Radiograph Dataset with Uncertainty Labels and Expert Comparison. Proc. AAAI Conf. Artif. Intell..

[86] Wang X., Peng Y., Lu L., Lu Z., Bagheri M., Summers R.M. ChestX-ray8: Hospital-scale Chest X-ray Database and Benchmarks on Weakly-Supervised Classification and Localization of Common Thorax Diseases. Proceedings of the IEEE Conference on Computer Vision and Pattern Recognition (CVPR).

[87] Weiner M.W., Veitch D.P., Aisen P.S., Beckett L.A., Cairns N.J., Green R.C., Harvey D., Jack C.R., Jagust W., Liu E. (2015). The Alzheimer’s Disease Neuroimaging Initiative: A review of papers published since its inception. Alzheimer’s Dement..

[88] Menze B.H., Jakab A., Bauer S., Kalpathy-Cramer J., Farahani K., Kirby J., Burren Y., Porz N., Slotboom J., Wiest R. (2014). The multimodal brain tumor image segmentation benchmark (BRATS). IEEE Trans. Med. Imaging.

[89] Baid U., Ghodasara S., Mohan S., Bilello M., Calabrese E., Colak E., Farahani K., Kalpathy-Cramer J., Kitamura F.C., Pati S. (2021). The rsna-asnr-miccai brats 2021 benchmark on brain tumor segmentation and radiogenomic classification. arXiv.

[90] Cassidy B., Kendrick C., Brodzicki A., Jaworek-Korjakowska J., Yap M.H. (2022). Analysis of the ISIC image datasets: Usage, benchmarks and recommendations. Med. Image Anal..

[91] Bejnordi B.E., Veta M., Van Diest P.J., Van Ginneken B., Karssemeijer N., Litjens G., Van Der Laak J.A., Hermsen M., Manson Q.F., Balkenhol M. (2017). Diagnostic assessment of deep learning algorithms for detection of lymph node metastases in women with breast cancer. JAMA.

[92] Underwood T. (2020). Pan-cancer analysis of whole genomes. Nature.

[93] Johnson A.E., Pollard T.J., Shen L., Lehman L.w.H., Feng M., Ghassemi M., Moody B., Szolovits P., Anthony Celi L., Mark R.G. (2016). MIMIC-III, a freely accessible critical care database. Sci. Data.

[94] Johnson A.E., Bulgarelli L., Shen L., Gayles A., Shammout A., Horng S., Pollard T.J., Hao S., Moody B., Gow B. (2023). MIMIC-IV, a freely accessible electronic health record dataset. Sci. Data.

[95] Pollard T.J., Johnson A.E., Raffa J.D., Celi L.A., Mark R.G., Badawi O. (2018). The eICU Collaborative Research Database, a freely available multi-center database for critical care research. Sci. Data.

[96] Wagner P., Strodthoff N., Bousseljot R.D., Kreiseler D., Lunze F.I., Samek W., Schaeffter T. (2020). PTB-XL, a large publicly available electrocardiography dataset. Sci. Data.

[97] Obeid I., Picone J. (2016). The temple university hospital EEG data corpus. Front. Neurosci..

[98] Weinstein J.N., Collisson E.A., Mills G.B., Shaw K.R., Ozenberger B.A., Ellrott K., Shmulevich I., Sander C., Stuart J.M. (2013). The cancer genome atlas pan-cancer analysis project. Nat. Genet..

[99] Lonsdale J., Thomas J., Salvatore M., Phillips R., Lo E., Shad S., Hasz R., Walters G., Garcia F., Young N. (2013). The genotype-tissue expression (GTEx) project. Nat. Genet..

[100] Bycroft C., Freeman C., Petkova D., Band G., Elliott L.T., Sharp K., Motyer A., Vukcevic D., Delaneau O., O’Connell J. (2018). The UK Biobank resource with deep phenotyping and genomic data. Nature.

[101] Regev A., Teichmann S.A., Lander E.S., Amit I., Benoist C., Birney E., Bodenmiller B., Campbell P., Carninci P., Clatworthy M. (2017). The human cell atlas. elife.

[102] Hasanah U., Leu J.S., Avian C., Azmi I., Prakosa S.W. (2025). A systematic review of multilabel chest X-ray classification using deep learning. Multimed. Tools Appl..

[103] Bhusal D., Panday S.P. (2022). Multi-label classification of thoracic diseases using dense convolutional network on chest radiographs. arXiv.

[104] Sammani F., Joukovsky B., Deligiannis N. (2023). Visualizing and understanding contrastive learning. IEEE Trans. Image Process..

[105] Chaitanya K., Erdil E., Karani N., Konukoglu E. (2020). Contrastive learning of global and local features for medical image segmentation with limited annotations. Adv. Neural Inf. Process. Syst..

[106] Ciga O., Xu T., Martel A.L. (2022). Self supervised contrastive learning for digital histopathology. Mach. Learn. Appl..

[107] Guo Z., Zhang Y., Qiu Z., Dong S., He S., Gao H., Zhang J., Chen Y., He B., Kong Z. (2023). An improved contrastive learning network for semi-supervised multi-structure segmentation in echocardiography. Front. Cardiovasc. Med..

[108] Luo G., Xie W., Gao R., Zheng T., Chen L., Sun H. (2023). Unsupervised anomaly detection in brain MRI: Learning abstract distribution from massive healthy brains. Comput. Biol. Med..

[109] Krishnan R., Rajpurkar P., Topol E.J. (2022). Self-supervised learning in medicine and healthcare. Nat. Biomed. Eng..

[110] Pick F., Xie X., Wu L.Y. (2024). Contrastive Multitask Transformer for Hospital Mortality and Length-of-Stay Prediction. Proceedings of the International Conference on AI in Healthcare.

[111] Nayebi Kerdabadi M., Hadizadeh Moghaddam A., Liu B., Liu M., Yao Z. Contrastive learning of temporal distinctiveness for survival analysis in electronic health records. Proceedings of the 32nd ACM International Conference on Information and Knowledge Management.

[112] Liu Y., Zhang Z., Qin S., Salim F.D., Yepes A.J. (2023). Contrastive learning-based imputation-prediction networks for in-hospital mortality risk modeling using ehrs. Proceedings of the Joint European Conference on Machine Learning and Knowledge Discovery in Databases.

[113] Zang C., Wang F. Scehr: Supervised contrastive learning for clinical risk prediction using electronic health records. Proceedings of the IEEE International Conference on Data Mining.

[114] Sun M., Yang X., Niu J., Gu Y., Wang C., Zhang W. (2024). A cross-modal clinical prediction system for intensive care unit patient outcome. Knowl.-Based Syst..

[115] Cai T., Huang F., Nakada R., Zhang L., Zhou D. (2024). Contrastive Learning on Multimodal Analysis of Electronic Health Records. arXiv.

[116] Zhong X., Batmanghelich K., Sun L. (2024). Enhancing Biomedical Multi-modal Representation Learning with Multi-scale Pre-training and Perturbed Report Discrimination. Proceedings of the 2024 IEEE Conference on Artificial Intelligence (CAI).

[117] Liu X., Xu X., Xu X., Li X., Xie G. Representation Learning for Multi-omics Data with Heterogeneous Gene Regulatory Network. Proceedings of the 2021 IEEE International Conference on Bioinformatics and Biomedicine (BIBM).

[118] Li S., Ma J., Zhao T., Jia Y., Liu B., Luo R., Huang Y. (2024). CellContrast: Reconstructing spatial relationships in single-cell RNA sequencing data via deep contrastive learning. Patterns.

[119] Bepler T., Berger B. (2021). Learning the protein language: Evolution, structure, and function. Cell Syst..

[120] Zhang R., Wu H., Liu C., Li H., Wu Y., Li K., Wang Y., Deng Y., Chen J., Zhou F. (2024). Pepharmony: A multi-view contrastive learning framework for integrated sequence and structure-based peptide encoding. arXiv.

[121] Huang W., Li C., Zhou H.Y., Yang H., Liu J., Liang Y., Zheng H., Zhang S., Wang S. (2024). Enhancing representation in radiography-reports foundation model: A granular alignment algorithm using masked contrastive learning. Nat. Commun..

[122] Koleilat T., Asgariandehkordi H., Rivaz H., Xiao Y. (2024). Medclip-sam: Bridging text and image towards universal medical image segmentation. Proceedings of the International Conference on Medical Image Computing and Computer-Assisted Intervention.

[123] Liu Z., Alavi A., Li M., Zhang X. (2023). Self-supervised contrastive learning for medical time series: A systematic review. Sensors.

[124] Yuan Y., Van Duyn J., Yan R., Huang Z., Vesal S., Plis S., Hu X., Kwak G.H., Xiao R., Fedorov A. (2025). Learning ECG Representations via Poly-Window Contrastive Learning. arXiv.

[125] Wang Y., Han Y., Wang H., Zhang X. (2023). Contrast everything: A hierarchical contrastive framework for medical time-series. Adv. Neural Inf. Process. Syst..

[126] Raghu A., Chandak P., Alam R., Guttag J., Stultz C. Contrastive pre-training for multimodal medical time series. Proceedings of the NeurIPS 2022 Workshop on Learning from Time Series for Health.

[127] Guo H., Xu X., Wu H., Liu B., Xia J., Cheng Y., Guo Q., Chen Y., Xu T., Wang J. (2025). Multi-scale and multi-modal contrastive learning network for biomedical time series. Biomed. Signal Process. Control.

[128] Sun X., Yang Y., Dong X. (2025). Enhancing Contrastive Learning-based Electrocardiogram Pretrained Model with Patient Memory Queue. arXiv.

[129] Zech J.R., Badgeley M.A., Liu M., Costa A.B., Titano J.J., Oermann E.K. (2018). Variable generalization performance of a deep learning model to detect pneumonia in chest radiographs: A cross-sectional study. PLoS Med..

[130] Oakden-Rayner L., Dunnmon J., Carneiro G., Ré C. Hidden stratification causes clinically meaningful failures in machine learning for medical imaging. Proceedings of the ACM Conference on Health, Inference, and Learning.

[131] Guo C., Pleiss G., Sun Y., Weinberger K.Q. (2017). On calibration of modern neural networks. Proceedings of the International Conference on Machine Learning.

[132] Mehrabi N., Morstatter F., Saxena N., Lerman K., Galstyan A. (2021). A survey on bias and fairness in machine learning. ACM Comput. Surv. (CSUR).

[133] Samek W., Wiegand T., Müller K.R. (2017). Explainable Artificial Intelligence: Understanding, Visualizing and Interpreting Deep Learning Models. arXiv.

[134] Ribeiro M.T., Singh S., Guestrin C. “Why should I trust you?” Explaining the predictions of any classifier. Proceedings of the 22nd ACM SIGKDD International Conference on Knowledge Discovery and Data Mining.

[135] Selvaraju R.R., Cogswell M., Das A., Vedantam R., Parikh D., Batra D. Grad-CAM: Visual Explanations from Deep Networks via Gradient-Based Localization. Proceedings of the IEEE International Conference on Computer Vision (ICCV).

[136] Finlayson S.G., Bowers J.D., Ito J., Zittrain J.L., Beam A.L., Kohane I.S. (2019). Adversarial attacks on medical machine learning. Science.

[137] Arjovsky M., Bottou L., Gulrajani I., Lopez-Paz D. (2019). Invariant Risk Minimization. arXiv.

[138] Schölkopf B., Locatello F., Bauer S., Ke N.R., Kalchbrenner N., Goyal A., Bengio Y. (2021). Toward Causal Representation Learning. Proc. IEEE.

[139] McMahan H.B., Moore E., Ramage D., Hampson S., y Arcas B.A. (2017). Communication-Efficient Learning of Deep Networks from Decentralized Data. Proceedings of the 20th International Conference on Artificial Intelligence and Statistics (AISTATS).

[140] Abadi M., Chu A., Goodfellow I., McMahan H.B., Mironov I., Talwar K., Zhang L. (2016). Deep Learning with Differential Privacy. Proceedings of the 2016 ACM SIGSAC Conference on Computer and Communications Security (CCS).

[141] Rajpurkar P., Chen E., Banerjee O., Topol E.J. (2022). AI in health and medicine. Nat. Med..

